# Crystal Structures Reveal Hidden Domain Mechanics in Protein Kinase A (PKA)

**DOI:** 10.3390/biology12111370

**Published:** 2023-10-26

**Authors:** Colin L. Welsh, Abigail E. Conklin, Lalima K. Madan

**Affiliations:** 1 Department of Cellular and Molecular Pharmacology and Experimental Therapeutics, College of Medicine, Medical University of South Carolina, Charleston, SC 29425, USA; 2 Hollings Cancer Center, Medical University of South Carolina, Charleston, SC 29425, USA

**Keywords:** protein kinases, protein kinase (A), kinase structure, catalytic domain, structural dynamics, crystal B-factors

## Abstract

**Simple Summary:**

Understanding the changes that occur in proteins as they perform their cellular function is critical to understanding our biology. Here, we investigated PKA, a protein partially responsible for processing signals that allows cells to respond to their environment, as well as how the shape of this protein changes as it performs its role by chemically altering other proteins. We analyzed a repertoire of publicly available crystal structures of PKA and identified regions of PKA that change shape based on its physical interactions with its ligands, or other signaling proteins. Studies of these shape changes allow researchers to better explore PKA for making advances in improving existing pharmacological therapies and gaining a deeper understanding of disease biology.

**Abstract:**

Cyclic-AMP-dependent protein kinase A (PKA) is a critical enzyme involved in various signaling pathways that plays a crucial role in regulating cellular processes including metabolism, gene transcription, cell proliferation, and differentiation. In this study, the mechanisms of allostery in PKA were investigated by analyzing the vast repertoire of crystal structures available in the RCSB database. From existing structures of murine and human PKA, we elucidated the conformational ensembles and protein dynamics that are altered in a ligand-dependent manner. Distance metrics to analyze conformations of the G-loop were proposed to delineate different states of PKA and were compared to existing structural metrics. Furthermore, ligand-dependent flexibility was investigated through normalized B′-factors to better understand the inherent dynamics in PKA. The presented study provides a contemporary approach to traditional methods in engaging the use of crystal structures for understanding protein dynamics. Importantly, our studies provide a deeper understanding into the conformational ensemble of PKA as the enzyme progresses through its catalytic cycle. These studies provide insights into kinase regulation that can be applied to both PKA individually and protein kinases as a class.

## 1. Introduction

Protein kinases are a family of enzymes that assist in a multitude of signaling processes within cells, catalyzing protein phosphorylation to activate or inhibit cellular proteins [1]. These enzymes transfer the gamma-phosphate of ATP to target Ser/Thr/Tyr residues of proteins and alter their surface charges to affect their protein–protein interactions [2]. Most kinases function in signaling cascades that connect and amplify signaling from growth hormone receptors and neurotransmitters to bring changes in specific gene transcription and translation [3]. Modulation in kinase activity by deletion, mutations, gene amplification, or changes in regulation is linked to various inflammatory, neurological, cardiovascular, metabolic, and cancerous conditions [4]. Understandably, protein kinases continue to be important therapeutic targets for combating various human diseases [5].

The cyclic-AMP-dependent protein kinase A (PKA) was the first eukaryotic kinase to be identified and structurally explored [6,7]. PKA has therefore been used as a model for protein kinase research. Studies on PKA activation, allosteric regulation, and dynamic interaction with substrates continue to enhance our comprehension of protein kinase biochemistry and its associated cell signaling. As a signaling complex (or signalosome), PKA is a holoenzyme tetramer of two catalytic (C)-subunits (protein kinase component) and two regulatory (R)-subunits (cyclic nucleotide binding component) docked to membranes or organelles by anchoring proteins called AKAPs [6,8,9,10,11]. These respond to G-proteins and growth factor receptor activation and transmit the signal to downstream proteins. Briefly, hormone binding to growth factor receptors mediates the activation of adenylate cyclases that convert ATP to cAMP. Increased levels of cAMP bind the R-subunits on the PKA holoenzymes and disrupt them to release the C-subunits [8]. The free C-subunits phosphorylate substrate proteins in the cytosol, or travel to the nucleus by binding to interactors like PKI [12,13,14,15,16,17]. In the nucleus, the C-subunit phosphorylates transcription factors including CREB and alters the expression of target genes [18,19]. In this article, we utilized the expansive database of PKA C-subunit structures to analyze dynamic details and mechanistic properties of the conserved kinase domain. Our studies provide a fresh approach to analyzing protein crystal structures for dynamic information and curating molecular properties of kinases that can be leveraged for further investigations.

**Figure 1:**
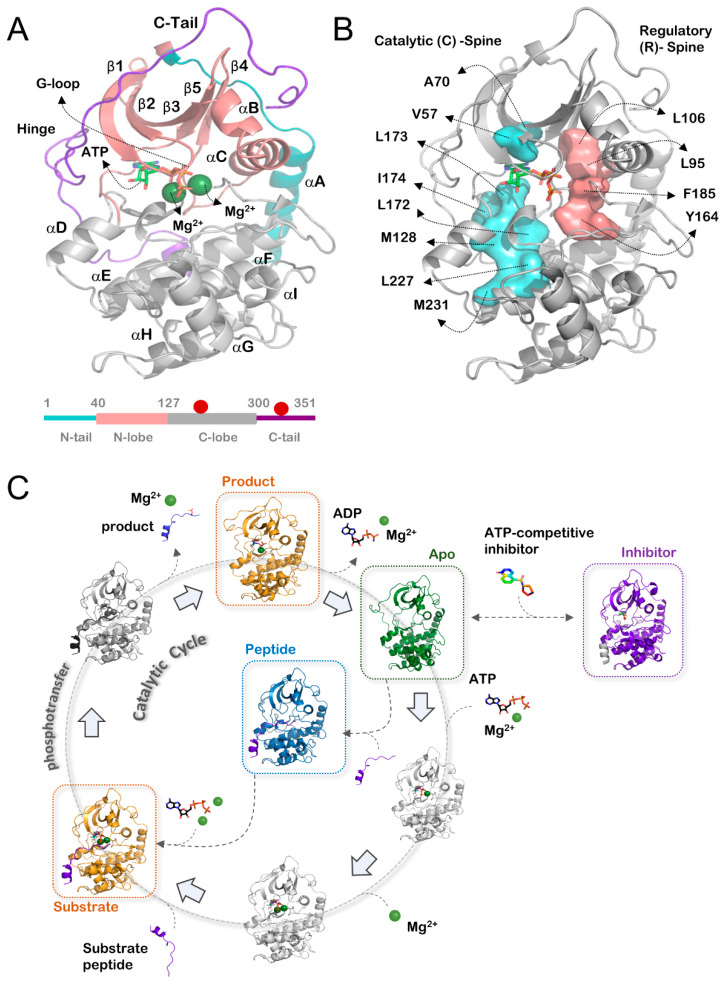
(**A**) Top, structure of PKA (PDB ID: 1ATP) and secondary structure features. Structure is colored by region: N-tail (cyan), N-lobe (salmon), C-lobe (gray), C-tail (purple). Bottom, domain architecture of PKA. Phosphorylation sites are noted as red circles. (**B**) Location of hydrophobic spine residues on the structure of PKA (PDB ID: 1ATP). Catalytic (C)-spine (cyan), regulatory (R)-spine (salmon). (**C**) Catalytic cycle of PKA. Defined states in this study are highlighted in color: Substrate/product (PKA_S/P_) (orange), inhibitor (PKA_inhibitor_) (purple), apo (PKA_apo_) (green), peptide only (PKA_peptide_) (blue).

The PKA C-subunit (henceforth only PKA) includes the kinase core (residues 40–300) flanked by two tails: an N-terminal tail (residues 1–40) and a C-terminal tail (residues 300–350) (Figure 1A) [20,21,22]. The kinase core is a bi-lobal structure with the N-lobe and C-lobe, connected by a flexible hinge [6]. The kinase active site is at the cleft between the two lobes. The smaller N-lobe has a central b-sheet flanked by a mobile αC helix and contains the invariant K72 required for binding ATP and two divalent metal ions (Mg^2+^ or Mn^2+^) [23,24]. A conserved E91 from the αC helix makes an invariant salt bridge with K72. The larger C-lobe is mostly α-helical and contains two central αE and αF helices that form the most conserved region of the kinase core. The C-lobe contains the peptide-binding cleft to recruit incoming substrate Ser/Thr regions on proteins/peptides. Dynamic movements of the two lobes allow for the opening/closing of the kinase active site and access to ATP and substrates [25]. Crystal structures reveal three conformations: “open”, “closed”, or “intermediate” classified by the relative distance and orientation of the N- and C-lobes. Open conformations display an increased inter-lobe distance, whereas closed conformations have the lobes closer to one another [20,21,23,26].

Important catalytic modules of the kinase domain include a glycine rich loop (G-loop) connecting the β1- and β2-strands of the N-lobe. This loop facilitates ATP binding and positioning at the active site [27]. The loop contains a GxGxxG consensus motif where the second and third glycine positions flank a regulatory phosphorylation site in some kinases (S35 in PKA) [28,29]. The active site contains a HRD motif (residues Y164, R165, D166 in PKA) that harbors the catalytic D166 required for phosphotransfer [30,31]. While the HRD motif is named based on the sequence similarity of the broader kinase family, the AGC kinases, including PKA, in fact have a Y-substitution at the first position. Despite PKA having residues Y164, R165, and D166 in these positions, we refer to this motif as the HRD motif to ensure consistency with other kinase literature. A Mg^2+^-binding loop containing a DFG motif (residues D184, F185, and G187 in PKA) coordinates ATP/divalent ions at the active site. Two autophosphorylation sites at positions T197 (in the activation loop) and S338 (in the C-terminal tail) allow for maintaining the “active”, catalytically competent state [32,33]. Phosphorylation of the activation loop pT197 arranges the catalytic and magnesium-binding loops in a catalytically conforming orientation. This pT197 also engages with a pH-sensitive H87 at the N-terminus of the αC-helix to facilitate optimal substrate binding at the kinase active site [34]. C199 of the activation segment makes pT197 resistant to hydrolysis by protein phosphatases [35].

Activation and optimal catalysis by the kinase domain are dynamically linked to the assembly of two hydrophobic “spines” in the kinase core (Figure 1B) [36]. The regulatory (R)-spine includes residues from the catalytic and magnesium-binding loops and is representative of the activation of the kinase domain. The R-spine includes four residues: RS1 from the HRD motif (Y164), RS2 from the DFG motif (F185), RS3 at a conserved aliphatic residue from the αC-helix (L95), and RS4 from the β4-strand (L106). Proper alignment of the R-spine is a prerequisite for kinase activation. In the absence of activation cues, like in the R194A mutant that evades pT197 phosphorylation, the R-spine is disassembled and PKA is inactive. The second hydrophobic spine is called the catalytic (C)-spine as it is completed upon ATP binding to the kinase domain and represents the catalytically competent conformation of PKA [37]. This spine includes residues from the ATP-binding cleft that regulate opening–closing motions between the two kinase lobes as it passes through its catalytic cycle [38,39,40].

A typical PKA catalytic cycle works through a “linchpin” mechanism where the two divalent metal ions (usually Mg^2+^) balance electrostatic charges at the active site and facilitate ADP release (Figure 1C) [41]. Catalysis begins with PKA binding ATP bound to a single Mg^2+^ (M1) at the high-affinity site. This is followed by substrate binding and recruitment of another Mg^2+^ (M2) at the low-affinity site [42]. Several PKA residues (K168, E91, H87, K72, Y204) stabilize a catalytically competent ternary complex and synchronize ATP/2 Mg^2+^ with the substrate hydroxyl-side chains [23,38]. Phosphotransfer takes place via a transition state between the ATP and hydroxyl ions while utilizing D166 of PKA as the general acid, and D184 and N171 as the stabilizers [43]. Certain hybrid quantum mechanics/molecular mechanics calculations hint towards a catalytic role for M2 as a Lewis base, attacking the b-g bridging oxygen of ATP to facilitate phosphotransfer [43,44,45,46,47,48,49,50]. Following catalysis, the phosphorylated Ser/Thr peptide leaves the kinase active site. At the same time, the “linchpin” M2 also exits the kinase active site and creates a charge imbalance to promote ADP-release [41]. In the final step of the catalytic cycle, ADP bound to M1 exits the kinase site to facilitate the next turnover cycle. This last step of ADP-release is the rate-determining step in the catalytic cycle and accounts for PKA’s turnover (k_cat_) of 20 s^−1^ [51].

The regulation of PKA’s activity through protein dynamics and conformational changes has been a topic of much consideration. The most notable regulatory mechanisms concern two features: the DFG motif and αC helix. These undergo conformational changes that impact the catalytic activity of the kinase domain. The αC helix is able to undergo a variety of motions but can be described generally by two conformations: αC-in, defined by a conserved salt bridge between the β3 lysine and αC glutamate residues, and αC-out, defined by the absence of this salt bridge and the displacement of the αC helix [52,53]. The swinging-in and out motion of the αC helix is modulated by a pH-sensitive salt bridge between its N-terminus H87 residue and the pT197 phospho-site in the kinase activation segment [34]. The DFG motif has a more varied ensemble, being able to adopt a variety of conformations that can be well defined by the dihedral angles of the phenylalanine sidechain. These states consist of DFG_in_, the typical active conformation of kinases; DFG_out_, where the phenylalanine sidechain occupies the ATP binding pocket and the kinase is inactive; and DFG_inter_, a variety of conformations that lie between the two aforementioned states [54].

By examining C-subunits across different PKA structures, differences in key parts of the conformational ensemble can reveal the mechanisms behind protein kinase activity. To elucidate these subtle conformational changes, the C-subunit was evaluated across 115 structures to determine the different conformations across stages of PKA’s catalytic cycle. Distances in chosen residues were taken to determine how different residues, loops, spines, and helices across structures changed in open, closed, and intermediate states. Through this analysis of existing crystal structures, a deeper understanding of protein dynamics and flexibility is achieved, helping to display PKA residue integration and how the enzyme transitions between its distinct ligand-bound states.

## 2. Methods

### 2.1. Data Set Compilation and Pair-Wise Distance Analysis

Crystal structures of human and murine PKA were accessed from the RCSB PDB [55] and assigned a state based on the bound nucleotide/inhibitor. A complete list of structures analyzed is provided in the supplementary tables (Tables S1, S2, S5 and S6). Structures were grouped into apo (no nucleotide or peptide bound), peptide bound (only peptide bound), substrate/product (ATP/ADP with/without peptide bound), and inhibitor (non-hydrolysable AMP-PNP/other Type I-ATP competitive inhibitor with/without peptide bound) categories based on the ligand occupying the kinase active site. For structures with multiple PKA molecules in the asymmetric unit (AU), distances were measured for all molecules and included in the dataset as individual entries. Distances between residues were measured as the distance between the Cs of those residues, unless otherwise noted. Measurements were performed in PyMOL [56], and data were visualized/analyzed in matplotlib [57] and seaborn [58].

### 2.2. B′-Factor Analysis

The normalization of protein B-factors was performed as described elsewhere [59,60]. B-factors were acquired from structure files through Biopython [61]. For an atom *i* with multiple defined positions, the B-factor was defined as a function of the occupancy *π* of all alternate positions *I*.

$$B\left(i\right)=\sum \pi \left(I\right)B(i,I)$$

Outliers were excluded from analysis through a median-based method, where the B-factor for atom *B*(*i*) was included based on the values of the median B-factor $\tilde{B}$ and the median absolute deviation *MAD*.

$$MAD=median\left[\sqrt{{\left(B\left(i\right)-\tilde{B}\right)}^{2}}\right]$$

$$M\left(i\right)=\frac{0.6745\cdot \left(B\left(i\right)-\tilde{B}\right)}{MAD}$$

An *M*(*i*) > 3.5 was used as the cutoff for exclusion of the value. Once outliers were excluded from the dataset, the normalized values *B*′(*i*) were calculated from the mean $\bar{B}$ and standard deviation *σ*.

$$B^{\prime }\left(i\right)=\frac{B\left(i\right)-\bar{B}}{\sigma }$$

For a per-residue analysis, the normalized value for residue *i* with atoms *a*, varying between all-atom, backbone, and C_α_ depending on analysis, was weighted by the molecular weight *M* of the component atoms and residue as a whole.

$$B^{\prime }\left(i\right)=\frac{1}{M(i)}\sum M\left(a\right)B^{\prime }(a)$$

The per-residue B′-factors were used for further analysis. The per-residue mean and 95% confidence intervals were calculated in seaborn [58]. Principal component analysis (PCA) of the B′-factors was performed using scikit-learn [62]. For structures with multiple PKA molecules in the AU, only the first molecule in the structure was included in the PCA calculation, according to the chain names assigned by the PDB.

## 3. Results

### 3.1. Catalytic States of PKA

The catalytic cycle of PKA is comprised of a multiple step mechanism, containing numerous binding and dissociation events in addition to the phosphotransfer of the γ-phosphate from ATP to a substrate peptide (Figure 1C). These processes are associated with conformational changes, including both larger motions of the bi-lobal structure as well as subtle changes in loop conformations. To examine the dependence of kinase conformation on different types of ligands, we performed a structural analysis of the crystal structures of murine (*n* = 65) and human (*n* = 50) PKA, classifying the structures into four different states:

- Substrate/product (*n* = 37): PKA bound to ATP or ADP, with or without a substrate peptide.
- Inhibitor (*n* = 60): PKA bound to a small molecule that is unable to be used for phosphotransfer (e.g., AMP-PNP or Type-I inhibitors).
- Apo (*n* = 15): PKA without a small molecule or peptide in the active site.
- Peptide (*n* = 3): PKA without a small molecule in the active site and with a peptide bound.

### 3.2. Ligand-Induced Closing of the N- and C-Lobes

**Figure 2:**
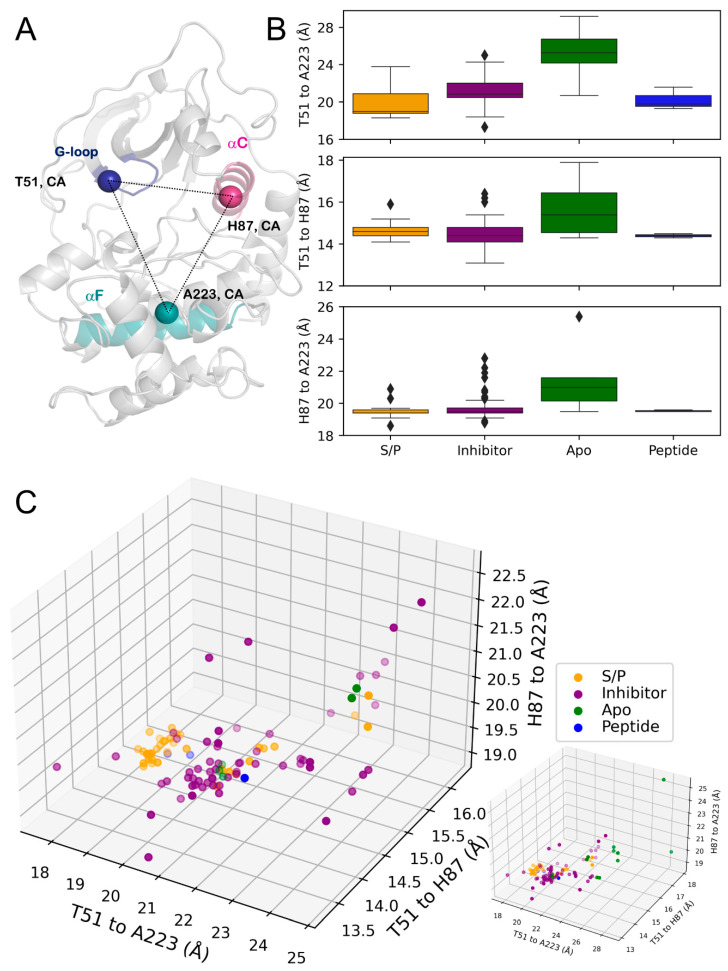
(**A**) Analyzed distances on the structure of PKA (PDB ID: 1ATP) with secondary structure locations colored: G-loop (blue), αC helix (pink), αF helix (turquoise). (**B**) Box-and-whisker plots of C_α_-C_α_ distances. Top, T51-A223. Middle, T51-H87. Bottom, H87-A223. (**C**) Three-dimensional scatterplot of distances. Left, zoomed plot showing main clustering of points. Bottom right, full plot including outliers. The transparency of points indicates their depth on the plot for visualization purposes.

The open/closed conformations of PKA are directly correlated with the enzyme’s activity, as the lobes must be closer together in the “closed” conformation for efficient phosphotransfer. To investigate the role of substrates on this conformational change, we measured C_α_-C_α_ distances of three residues: T51 and H87 on the N-lobe and A223 on the C-lobe (Figure 2A, Table 1). These residues specifically detail motions seen between the G-loop (T51), the aC helix (H87) on the N-lobe, and the buried/immobile aF helix (A223) on the C-lobe [23]. Comparing the four states of PKA investigated in this study, PKA_apo_ displayed an increase in the mean distance between all three residues (Figure 2B, Table 1). Between the substrate/product- and inhibitor-bound states, the divergence was more subtle. The T51-A223 distance was where the difference between the two states was most apparent, in which the PKA_inhibitor_ showed an increase in the mean distance of these two residues.

Plotting these distances together, the majority of different states of PKA clustered separately, with some overlap (Figure 2C, Table 1). Interestingly, some PKA_apo_ structures displayed conformations more similar to the inhibitor-bound state. Furthermore, several PKA_S/P_ structures grouped around a T51-A223 distance of ≈21Å, similar to the majority of PKA_inhibitor_ structures, albeit with an increased T51-H87 distance. The minimum distance and first quartile of distance distribution of PKA_apo_ matched the third quartile and maximum distances for the PKA_S/P_ and PKA_inhibitor_ states. These results indicate that the “closed” conformation was stabilized by ligand/substrate binding to the kinase active site while dynamics allowed the PKA_apo_ to span both the open and close states. Surprisingly, while nucleotide-and-peptide binding is known to follow positive cooperative kinetics [25,38], the PKA_Peptide_ structures showed similar “closed” conformations as seen in the ternary PKA_S/P_ complexes. As the number of structures of the PKA_Peptide_ state were limited for efficient statistical analysis, no conclusions could be drawn about the role of peptide alone on regulating the opening/closing dynamics of PKA.

In the present study, T51 was used to measure the interlobe distance instead of the previously used S53 residue [23] to deconvolute lobe opening/closing from inversions of the tip of the G-loop in certain inhibitor bound structures (Figure S1, Table S3). Using S53, H87 and A223 showed an increased number of outliers in the PKA_inhibitor_ group due to engagement of F54 of the G-loop with the inhibitor in these structures. Taking the S53 metric provided data indicating PKA_inhibitor_ group structures to span conformations that were “more closed” than the PKA_S/P_ complexes and even more “open” than the PKA_apo_ states. An inspection of these structures showed distortions in the G-loop that were unrelated to the conformation of the rest of the N-lobe.

### 3.3. PKA Preferred a DFG_in_ Conformation

**Figure 3:**
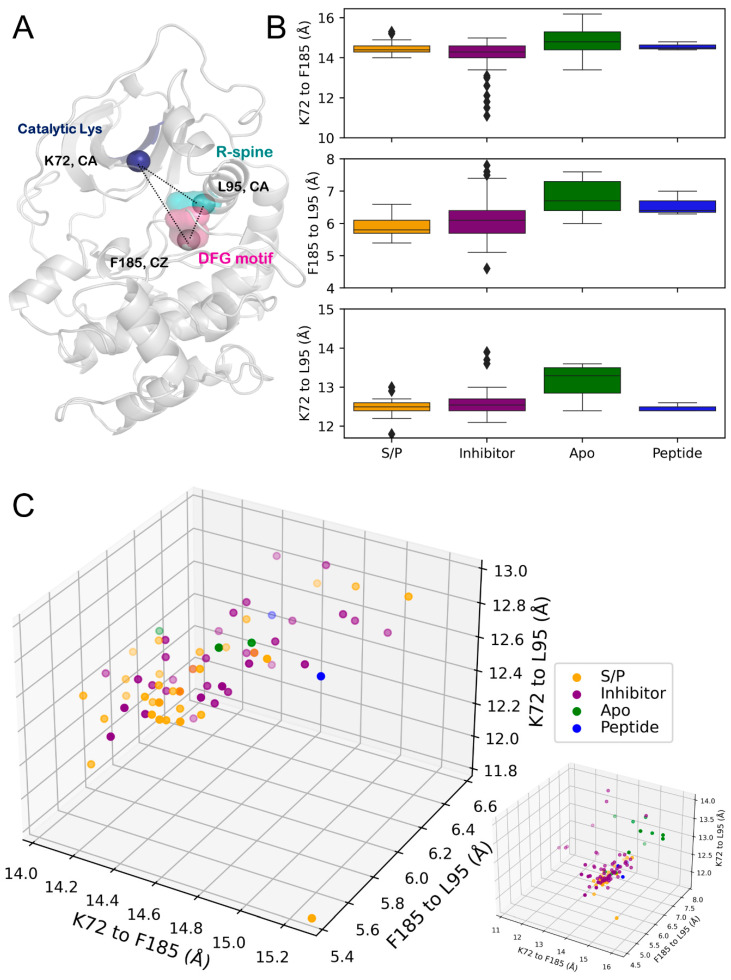
(**A**) Analyzed distances on the structure of PKA (PDB ID: 1ATP) with structural locations colored: β3 strand (blue), DFG motif (pink), R-spine (turquoise). (**B**) Box-and-whisker plots of distances. Top, K72_Cα_-F185_Cζ_. Middle, F185_Cζ_-L95_Cα_. Bottom, K72_Cα_-L95_Cα_ (**C**) Three-dimensional scatterplot of distances. Left, zoomed plot showing main clustering of points. Bottom right, full plot including outliers. The transparency of points indicates their depth on the plot for visualization purposes.

The orientation of the F185, one residue that comprises the DFG motif in kinases, has been identified as one metric to characterize the “activation” state of kinases [54]. This is defined as the distance between the C_ζ_ of the phenylalanine in DFG, F185 in PKA, to the C_α_ of the catalytic lysine, K72, or the C_α_ of the residue at the +4 position of the αC helix’s glutamate residue, in this case L95 (Figure 3A, Table 2). Here, residues F185 and L95 are RS2 and RS3 residues of the R-spine that connect the kinase N- and C-lobes [36]. Distance measurements between C_a_ atoms of K72, L95, and C_ζ_ of F185 provide a quantitative metric for the assembly of the R-spine and is reflective of the active state of the kinase structure [37].

In our analysis, nearly all of the analyzed structures fell into the definitions of DFG_in_, where the F185_Cζ_-K72_Cα_ distance distribution centered around 15Å, and the F185_Cζ_-L95_Cα_ distance distributed around ≈7Å^51^ (Figure 3B, Table 2). The distribution of structures across these three distances did not yield clearly clustered states, but there were trends that could be observed (Figure 3C, Table 2). PKA_S/P_ tends to have a decreased F185_Cζ_-L95_Cα_ distance but is not unique among states in that regard. The clearest separation present in this data set was the tendency of PKA_apo_ to have a ≈1Å increase in the third distance, the C_α_-C_α_ distance of K72 to L95, which clustered 8/11 of the PKA_apo_ structures. Several of the PKA_inhibitor_ structures approached the definition of DFG_inter_ due to a decrease in the F185_Cζ_-K72_Cα_ distance, but these were the few outliers in the distribution. Closer inspection of these outlier structures showed distortion in the N-lobe and active site cleft where the inhibitor bound hydrophobic residues to create an “less active” kinase conformation.

To further investigate the dynamics of the hydrophobic core of the kinase domain, we measured distances between the C_a_ atoms of V104 (core/shield [23] residue), K81 in the αB helix, and N113 in the b4-b5 loop (Figure S2, Table S4). PKA_inhibitor_ structures showed outliers for the V104-K81 distance indicative of structures with distorted/twisted the N-lobes with the bound inhibitors. No distinction could be made between the PKA_S/P_ and PKA_apo_ groups, indicating that this region of the N-lobe showed concerted movement in the opening/closing dynamics of the kinase domain.

### 3.4. G-Loop Conformation Was Sensitive to Ligand Type

**Figure 4:**
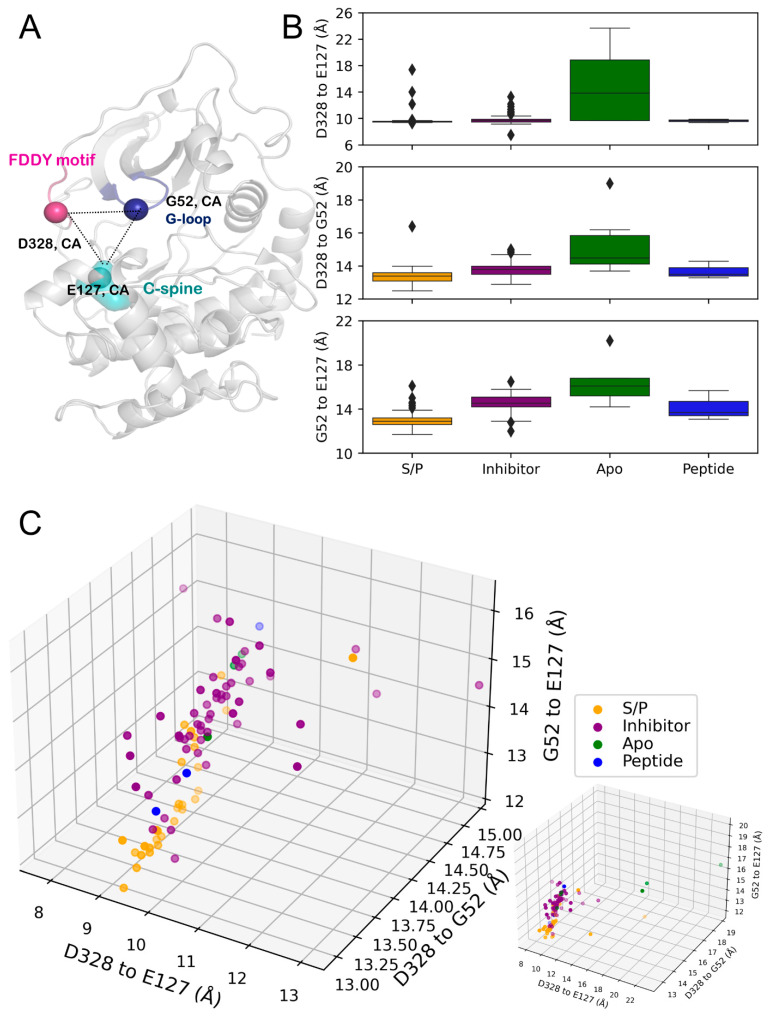
(**A**) Analyzed distances on the structure of PKA (PDB ID: 1ATP) with structural locations colored: G-loop (blue), FDDY motif (pink), C-spine (turquoise). (**B**) Box-and-whisker plots of C_α_-C_α_ distances. Top, D328-E127. Middle, D328-G52. Bottom, G52-E127. (**C**) Three-dimensional scatterplot of distances. Left, zoomed plot showing main clustering of points. Bottom right, full plot including outliers. The transparency of points indicates their depth on the plot for visualization purposes.

Another set of distances in this work investigated the conformations adopted by the G-loop as well as the C-tail of PKA. These residues included G52 of the G-loop, E127 of the catalytic spine residue, and D328 in the FDDY motif of the C-tail that contacts the adenine ring of ATP in the active site (Figure 4A, Table 3). As expected, several structures of PKA_apo_ did not contain a resolved D328 residue, and these were excluded from this portion of the analysis.

The apo form of PKA features increased C_α_-C_α_ distances for all three metrics, with the mean distance for D328-E127 displaying a ≈4 Å increase. This distance did not not display an increase between the substrate/product-, inhibitor-, or peptide-bound forms of PKA, indicating the role of ATP-binding in tethering the C-tail of PKA (Figure 4B, Table 3). However, the different conformations of the G-loop became apparent in a ligand-dependent manner. A small increase in the mean D328-G52 distance was seen between PKA_S/P_ and the inhibitor- or peptide-bound forms. This was mirrored in the G52-E127 mean distance, showing that the G-loop conformation was similarly affected by either inhibitor binding or the lack of ADP/ATP when bound to a peptide.

Investigating the distributions of these distances in a state-dependent manner, the similarity of the G-loop’s conformation between the peptide- and inhibitor-bound PKA became more apparent (Figure 4C, Table 3). PKA_apo_ showed divergence in these metrics, with structures appearing to distribute similarly to PKA_inhibitor_ but also with a dramatically increased D328-E127 distance due to conformational changes of the C-tail. PKA_S/P_ showed a split distribution, with one set of structures clustering independently, whereas others showed a G-loop conformation more similar to PKA_inhibitor_.

**Figure 5:**
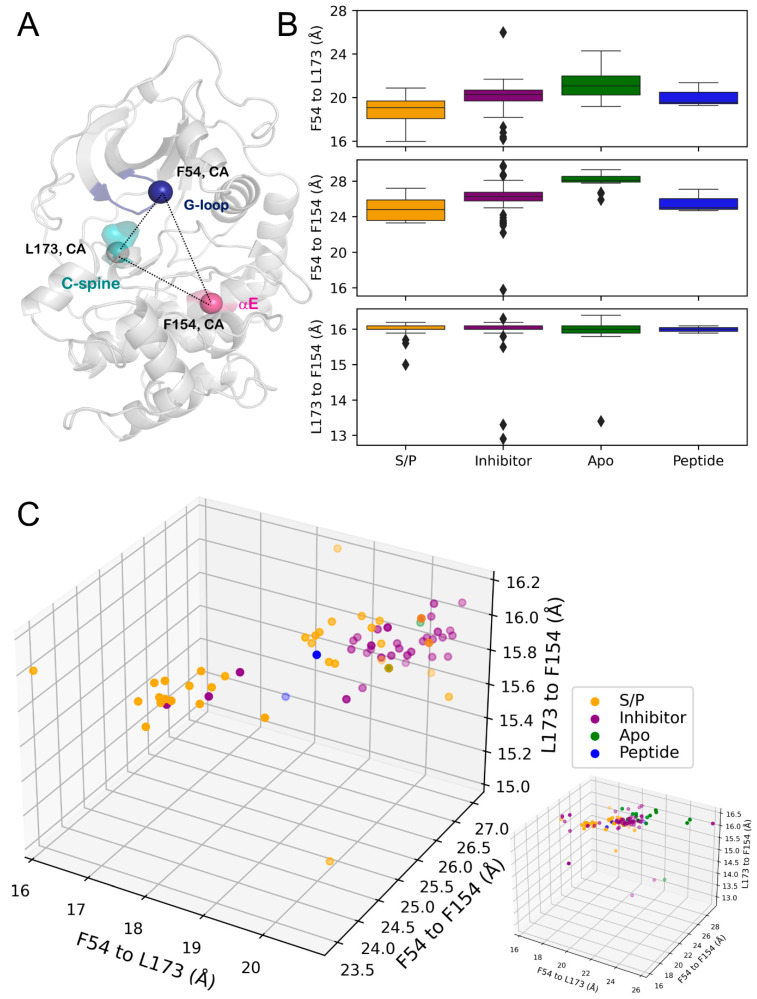
(**A**) Analyzed distances on the structure of PKA (PDB ID: 1ATP) with secondary structure locations colored: G-loop (blue), αE helix (pink), C-spine (turquoise). (**B**) Box-and-whisker plots of C_α_-C_α_ distances. Top, F54-L173. Middle, F54-F154. Bottom, L173-F154. (**C**) Three-dimensional scatterplot of distances. Left, zoomed plot showing main clustering of points. Bottom right, full plot including outliers. The transparency of points indicates their depth on the plot for visualization purposes.

To further investigate the conformational changes of the G-loop, distance measurements for residues F54, on the G-loop; F154, on the αE helix; and L173, a C-spine residue, were collected (Figure 5A, Table 4). This set of measurements supported the trends seen for ligand-dependent conformational changes of the G-loop, wherein PKA_S/P_ displayed a G-loop that was oriented more towards the active site of PKA.

This conformation was seen from the C_α_-C_α_ distances of F54-L173 and F54-F154, sharing the trend of PKA_S/P_ comprising the minimum mean distance (Figure 5B, Table 4). The structures for the PKA_inhibitor_ and PKA_peptide_ again displayed similar mean distances for these residue pairs. With some outliers, the C_α_-C_α_ distance for residues L173 and F154 remained nearly constant between states, indicating a conformational heterogeneity of PKA’s C-lobe. The split populations of the PKA_S/P_ G-loop were recapitulated when examining the distribution of structures across these three distances. One cluster of PKA_S/P_ clustered independently and was almost homogeneous, whereas the second cluster occupied a conformation more similar to the PKA_inhibitor_ structures (Figure 5C, Table 4).

### 3.5. B-Factor Variations Based on State

**Figure 6:**
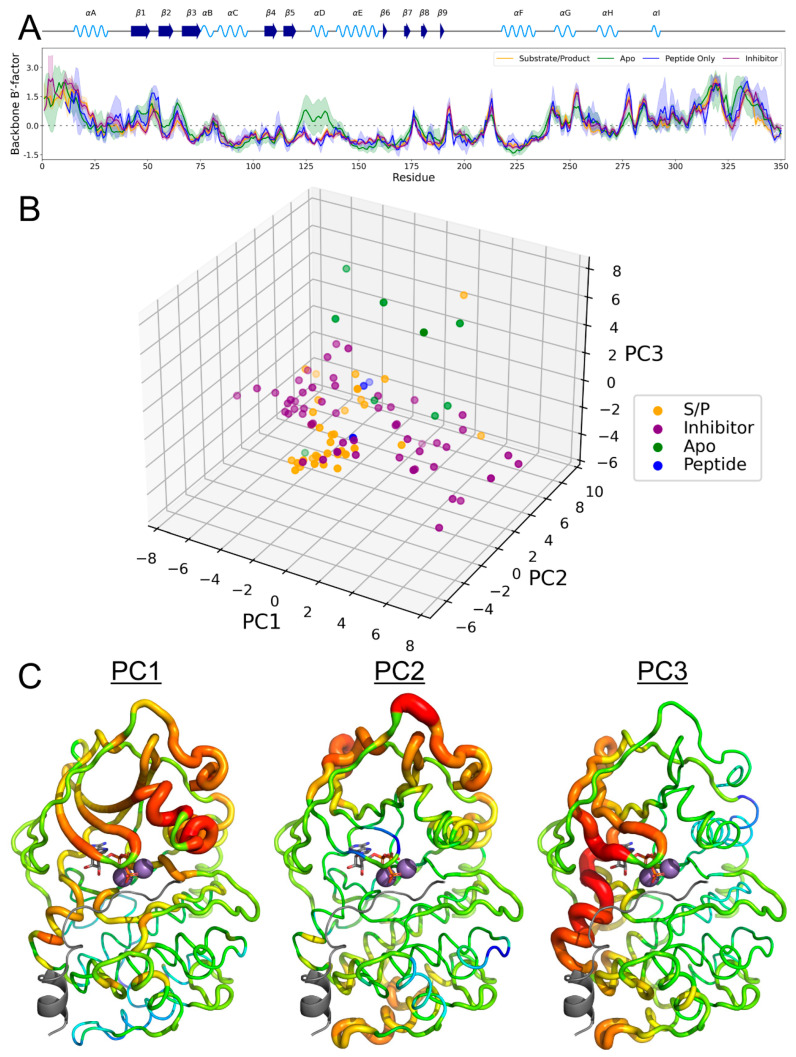
(**A**) Per-residue B′-factors of backbone atoms C_α_, N, C, and CO. Line indicates mean value, shadow indicates 95% confidence interval. Secondary structure of PKA is indicated above. (**B**) Three-dimensional scatterplot of each structure’s location on the first three PCs. (**C**) First three PCs mapped to the structure of PKA (PDB ID: 1ATP) in a B-factor putty representation. Increasing width and color progression from blue to red indicates increasing B′-factor. Peptide, ATP, and Mg^2+^ ions are shown in gray and do not have associated B′-factor values.

Through normalization of the B-factors of the included PKA crystal structures, the mean B′-factor and 95% confidence interval of the per-residue factors were calculated for the backbone atoms of each residue (Figure 6A). Interestingly, the B′-factors were strikingly similar between the different states of PKA. The most prominent difference between the Bʹ-factors was seen in the apo state, where the temperature factor was seen to increase in the αD helix and adjacent region. This phenomenon was also seen in analysis of the per-residue B′-factor using all heavy atoms or only C_α_ atoms (Figure S3).

PCA of the B′-factors allowed us to explore different PKA dynamics that were hidden in standard statistical analysis (Figure 6B). From the first three eigenvectors, the majority of the substrate/product-bound PKA formed an individual cluster, with the majority of structures occupying similar values along the first two eigenvectors. Inhibitor-bound PKA exhibited two populations along PC1, whereas the apo and peptide-bound forms showed a variety of values (with the exception of five apo PKA structures being distinguished along PC3). Determining the per-residue component of each eigenvector and mapping it to the structure yielded insights into state-specific temperature factor biases (Figure 6C). In PC1, in which a dual distribution exists for PKA_inhibitor_ and a single peak exists for PKA_S/P_, the stability of the protein decreased in the G-loop, as well as the flanking β1-β2 sheets, and the αC helix. Conversely, PC2 displayed an increased stability of the β1-G-loop-β2 feature. PC3 contains another increased motion of β1/β2 but had a unique decrease in stability of the αD helix similar to the PKA_apo_ state shown in Figure 6A.

### 3.6. PKA Mutants Changed Conformational Preference

**Figure 7:**
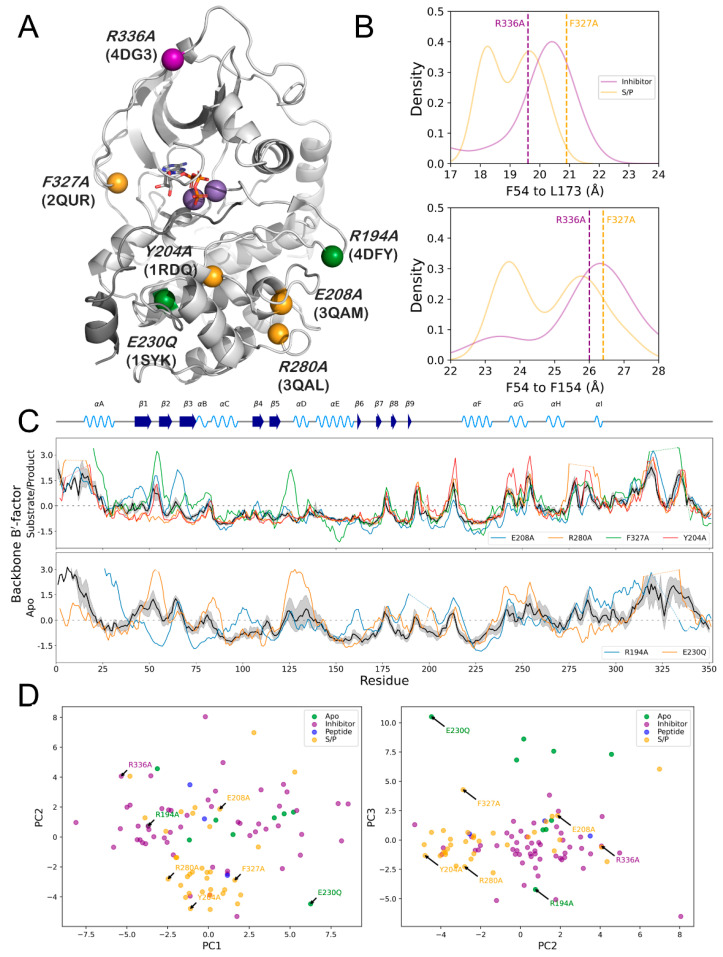
(**A**) Structural location, mutation, and PDB ID of PKA mutants included in the dataset on the structure of PKA (PDB ID: 1ATP). Color of the sphere indicates mutant state as defined in this study: substrate/product (orange), inhibitor (purple), apo (green). (**B**) Values for R336A (purple dashed line) and F327A (orange dashed line) compared to the kernel density estimation for the distance distributions of F54-L173 (top) and F54-F154 (bottom). (**C**) Backbone B′-factor values for different mutants in the substrate/product (top) or apo (bottom) states. Means and 95% confidence intervals for the entire per-state dataset are shown as black lines and gray shadows, respectively. (**D**) PKA mutant values for the first three PCs of the B′-factor PCA. Specific mutant values are indicated on the plot.

The structural data set of PKA used in this study included several mutant forms of the kinase, including mutations R194A (PDB ID: 4DFY) [32], Y204A (PDB ID: 1RDQ) [20], E208A (PDB ID: 3QAM) [63], E230Q (PDB ID: 1SYK) [64], R280A (PDB ID: 3QAL) [63], F327A/K285P (PDB ID: 2QUR) [65], and R336A (PDB ID: 4DG3) (unpublished) (Figure 7A, Table 5). These mutations were spread between the N- and C-lobes of PKA and consisted of four mutants in the substrate/product bound form, two mutants in the apo form, and one mutant in the inhibitor bound form.

Examining the effects of these mutations on the structure of PKA rationalizes their effects on the enzymatic activity of the kinase. Determining where these mutants fall on the probability distributions of the F54-L173 and F54-F154 measurements shows that mutations can induce PKA to adopt a state different than the typical ligand-dependent conformation (Figure 7B). In PKA_R336A_, an inhibitor-bound structure, the F54-L173 distance diverged from the peak density for that state and instead adopted the peak density conformation for the substrate/product bound state. Conversely, the PKA_F327A/K285P_ mutant showed the opposite effect: the F54-L173 distance for this mutant was more in line with the inhibitor probability distribution, as well as occupying the peak probability density conformation for the F54-F154 distance.

The B′-factors of the substrate/product state mutants R280A and F327A/K285P showed opposite effects on the temperature factor of specific regions of PKA. Both mutations showed perturbations to the B′-factor of the G-loop, located between the β1 and β2 sheets, but their effects were converse (Figure 7C). In PKA_F327A/K285P_, the G-loop showed an increased Bʹ-factor compared to the normalized mean, indicating a decrease in stability of this crucial active site loop. In PKA_R280A_, the mutation decreased the Bʹ-factor of this loop, showing an increase in stability compared to the normalized mean. Furthermore, the loop bridging the secondary structures β5 and αD showed an increase in B′-factor in PKA_F327A/K285P_, which is a region involved in binding the adenosine ring of ATP.

The apo PKA mutants R194A and E230Q displayed similar variance in their Bʹ-factor values of the G-loop. PKA_E230A_ showed a dramatic increase in the B′-factor compared to the mean normalized value, whereas PKA_R194A_ showed a decrease (Figure 7C). Additionally, the location of PKA_R194A_ in the distribution of the first three eigenvectors of the PCA indicated it is more similar to inhibitor-bound structures of PKA, despite it being in an unbound state (Figure 7D). This is in agreement with the PKA_R194A_ being an inactivating mutation.

## 4. Discussion

The current work presents a study of collated PKA structures to extract dynamic information based on different sets of distance triplets, defining subtle conformational changes induced by different stages in the catalytic cycle of the enzyme. The presented work adds to and advances the substantial research into defining active conformations of kinases as a class [66,67,68,69]. In addition to defined conformations of the DFG motif and the αC helix, we posit the G-loop as a further metric to define kinase activity where other metrics may not discern different states, as shown in our analysis of the DFG motif conformation (Figure 3). Particularly, a change in the conformation of the G-loop, important for ATP binding, was seen to change based on substrate/product or inhibitor binding, or in the absence of a ligand. This conformational change consisted of the tip of the G-loop, residues G52 and F54, shifting deeper into the ATP binding pocket (Figure 4 and Figure 5). In PKA_S/P_, these distances were at their respective minimums, with slight increases in the presence of an inhibitor. PKA_apo_ showed a drastic increase in these values, as well as greater variation in their distributions. Due to the conservation of the G-loop as well as its nature as a potential PTM site [28], this ligand-dependent conformational change is evidence for regulating the substrate specificity of kinases as a class.

The utility of B′-factor analysis in determining kinase conformation and state is shown in this study, albeit with limitations (Figure 6). The overall similarity of the B′-factors between states makes the metric a poor measurement of kinase activity. However, PCA of the B′-factors yielded individual components that separated different states of PKA. This shows the presence of state-specific B′-factors that can be used to further analysis kinase structures, but confounding factors from experimental details such as lattice disorder, crystal packing effects, and structure refinement technique impart substantial drawbacks on this analysis as a standalone metric [70]. With these caveats in mind, this analysis, or similar feature-extraction methods, can be a powerful tool to investigate kinase conformational ensembles.

Limitations of the presented study stem from the nature of the dataset and analysis performed. The dataset could certainly be expanded to include PKA from other species to increase the sample size, and further classification beyond four states could be performed to provide a finer-grained analysis of the structural ensemble of PKA. Additionally, future work should look to perform a more systematic analysis of pairwise distances to build upon the identified metrics presented here.

## 5. Conclusions

Through the analysis of PKA conformations based on several sets of distance triplets, we identified minute structural changes that show ligand dependency. The G-loop of PKA was shown to be particularly sensitive to ligand type and can be used as a basis for development of improved pharmacologic inhibitors of PKA. Overall, the present work further defines the conformational ensemble of PKA in a cellular environment, building on substantial work to elucidate the molecular mechanisms of kinase activity and regulation.

## Figures and Tables

**Table 1:** Measurement 1: Opening and closing dynamics of the kinase catalytic domain.

Distance (Å)	T51 (Ca) to A223 (Ca)	T51 (Ca) to H87 (Ca)	H87 (Ca) to A223 (Ca)
Substrate/Product			
Average	19.72	14.62	19.48
Median	19.00	14.60	19.40
Std Dev	1.48	0.34	0.34
N	37.00	37.00	37.00
Min Value	18.30	14.10	18.60
1st Quartile	18.80	14.40	19.40
2nd Quartile	19.00	14.60	19.40
3rd Quartile	20.90	14.80	19.60
Max Value	23.80	15.90	20.90
Inhibitor			
Average	21.24	14.54	19.76
Median	20.85	14.40	19.50
Std Dev	1.52	0.69	0.76
N	60.00	60.00	60.00
Min Value	17.30	13.10	18.80
1st Quartile	20.50	14.10	19.40
2nd Quartile	20.85	14.40	19.50
3rd Quartile	22.03	14.80	19.73
Max Value	25.00	16.40	22.80
Apo			
Average	25.15	15.59	21.31
Median	25.30	15.40	21.00
Std Dev	2.69	1.32	1.83
N	15.00	15.00	15.00
Min Value	20.70	14.30	19.50
1st Quartile	24.20	14.55	20.15
2nd Quartile	25.30	15.40	21.00
3rd Quartile	26.75	16.45	21.60
Max Value	29.20	17.90	25.40
Peptide			
Average	20.23	14.40	19.53
Median	19.80	14.40	19.50
Std Dev	1.21	0.10	0.06
N	3.00	3.00	3.00
Min Value	19.30	14.30	19.50
1st Quartile	19.55	14.35	19.50
2nd Quartile	19.80	14.40	19.50
3rd Quartile	20.70	14.45	19.55
Max Value	21.60	14.50	19.60

**Table 2:** Measurement 2: R-spine assembly in the kinase domain.

Distance (Å)	K72 (Ca) to F185 (Cz)	F185 (Cz) to L95 (Ca)	K72 (Ca) to L95 (Ca)
Substrate/Product			
Average	14.45	5.88	12.51
Median	14.40	5.80	12.50
Std Dev	0.27	0.29	0.19
N	37.00	37.00	37.00
Min Value	14.00	5.40	11.80
1st Quartile	14.30	5.70	12.40
2nd Quartile	14.40	5.80	12.50
3rd Quartile	14.60	6.10	12.60
Max Value	15.30	6.60	13.00
Inhibitor			
Average	14.12	6.16	12.62
Median	14.30	6.10	12.55
Std Dev	0.82	0.63	0.32
N	60.00	60.00	60.00
Min Value	11.10	4.60	12.10
1st Quartile	14.00	5.70	12.40
2nd Quartile	14.30	6.10	12.55
3rd Quartile	14.60	6.40	12.70
Max Value	15.00	7.80	13.90
Apo			
Average	14.91	6.81	13.14
Median	14.80	6.70	13.30
Std Dev	0.83	0.53	0.46
N	13.00	13.00	15.00
Min Value	13.40	6.00	12.40
1st Quartile	14.40	6.40	12.85
2nd Quartile	14.80	6.70	13.30
3rd Quartile	15.30	7.30	13.50
Max Value	16.20	7.60	13.60
Peptide			
Average	14.57	6.57	12.47
Median	14.50	6.40	12.40
Std Dev	0.21	0.38	0.12
N	3.00	3.00	3.00
Min Value	14.40	6.30	12.40
1st Quartile	14.45	6.35	12.40
2nd Quartile	14.50	6.40	12.40
3rd Quartile	14.65	6.70	12.50
Max Value	14.80	7.00	12.60

**Table 3:** Measurement 3: C-tail and G-loop dynamics.

Distance (Å)	D328 (Ca) to E127 (Ca)	D328 (Ca) to G52 (Ca)	G52 (Ca) to E127 (Ca)
Substrate/Product			
Average	9.95	13.44	13.12
Median	9.60	13.40	12.90
Std Dev	1.52	0.61	0.91
N	37.00	37.00	37.00
Min Value	9.30	12.50	11.70
1st Quartile	9.50	13.10	12.60
2nd Quartile	9.60	13.40	12.90
3rd Quartile	9.60	13.60	13.20
Max Value	17.40	16.40	16.10
Inhibitor			
Average	9.87	13.80	14.55
Median	9.70	13.80	14.55
Std Dev	0.81	0.50	0.80
N	58.00	58.00	58.00
Min Value	7.50	12.90	12.00
1st Quartile	9.50	13.50	14.20
2nd Quartile	9.70	13.80	14.55
3rd Quartile	9.90	14.00	15.10
Max Value	13.30	15.00	16.50
Apo			
Average	15.00	15.33	16.70
Median	13.85	14.50	16.10
Std Dev	6.08	2.00	2.13
N	6.00	6.00	9.00
Min Value	9.70	13.70	14.20
1st Quartile	9.73	14.13	15.20
2nd Quartile	13.85	14.50	16.10
3rd Quartile	18.88	15.85	16.80
Max Value	23.70	19.00	20.20
Peptide			
Average	9.67	13.70	14.17
Median	9.70	13.50	13.70
Std Dev	0.25	0.53	1.36
N	3.00	3.00	3.00
Min Value	9.40	13.30	13.10
1st Quartile	9.55	13.40	13.40
2nd Quartile	9.70	13.50	13.70
3rd Quartile	9.80	13.90	14.70
Max Value	9.90	14.30	15.70

**Table 4:** Measurement 4: C-spine and G-loop dynamics.

Distance (Å)	F54 (Ca) to L173 (Ca)	F54 (Ca) to F154 (Ca)	L173 (Ca) to F154 (Ca)
Substrate/Product			
Average	18.99	24.92	15.98
Median	19.20	25.30	16.00
Std Dev	0.98	1.23	0.21
N	33.00	33.00	33.00
Min Value	16.00	23.30	15.00
1st Quartile	18.20	23.60	16.00
2nd Quartile	19.20	25.30	16.00
3rd Quartile	19.70	25.90	16.10
Max Value	20.60	27.20	16.20
Inhibitor			
Average	20.08	25.94	15.94
Median	20.30	26.25	16.00
Std Dev	1.70	2.07	0.55
N	60.00	60.00	60.00
Min Value	16.20	15.80	12.90
1st Quartile	19.70	25.78	16.00
2nd Quartile	20.30	26.25	16.00
3rd Quartile	20.70	26.75	16.10
Max Value	26.00	29.70	16.30
Apo			
Average	20.12	24.48	15.14
Median	20.90	28.00	15.90
Std Dev	2.33	7.99	1.35
N	13.00	13.00	15.00
Min Value	15.30	6.60	13.00
1st Quartile	20.10	26.60	13.60
2nd Quartile	20.90	28.00	15.90
3rd Quartile	21.50	28.10	16.10
Max Value	22.50	29.00	16.40
Peptide			
Average	20.10	25.60	20.10
Median	19.60	25.00	19.60
Std Dev	1.14	1.31	1.14
N	3.00	3.00	3.00
Min Value	19.30	24.70	19.30
1st Quartile	19.45	24.85	19.45
2nd Quartile	19.60	25.00	19.60
3rd Quartile	20.50	26.05	20.50
Max Value	21.40	27.10	21.40

**Table 5:** Distance measurements as seen in mutant structures.

PDB	4DG3	3QAM	3QAL	2QUR	1RDQ	4DFY	4DFY	1SYK	1SYK
Chain ID	A	E	E	A	E	A	B	A	B
Mutants	*R336A*	*E208A*	*R280A*	*F327A*	*Y204A*	*R194A*	*R194A*	*E230A*	*E230A*
Distance (Å)	INHIBITOR	SUBSTRATE/PRODUCT				APO			
T51 to A223	18.9	19	18.8	22.6	18.5	27.4	27.4	29.2	29.2
T51 to H87	14.6	14.6	14.7	15.9	14.8	17.9	17.9	17.2	17.2
H87 to A223	19.5	19.3	19.4	19.7	19.3	25.4	25.4	20.7	20.7
D328 to E127	9.4	9.4	9.6	12.2	9.4	-	-	17.9	-
D328 to G52	13.3	12.9	13.1	14	12.9	-	-	16.2	-
G52 to E127	12.7	13.1	12.7	16.1	12.1	20.2	20.2	16	16.1
K72 to F185	14.4	14.2	14.4	14.6	14.5	-	-	15.3	15.3
F185 to L95	6.0	5.6	5.6	6.6	5.7	-	-	6.6	6.6
K72 to L95	12.6	12.4	12.6	12.7	12.5	13	13	13.6	13.6
F54 to L173	18.5	18.2	18.1	20.9	17.8	24.2	24.3	20.3	20.3
F54 to F154	27.1	23.6	23.6	26.4	23.4	29	29.3	28.2	28.2
L173 to F154	16.2	16	16	15.7	16	16	16.1	15.9	16
S53 to A223	19.5	19.5	19.2	23.5	18.7	28.1	27.9	28	28
S53 to H87	13.1	13.5	13.1	14.9	13.1	13.4	13.4	14.5	14.5
H87 to A223	19.5	19.3	19.4	19.7	19.3	25.4	25.4	20.7	20.7
K81 to V104	27.5	27.2	27	27.4	26.9	28.8	28.8	26.5	26.5
K81 to N113	16.6	16.4	16.5	16.4	16.4	16.4	16.5	16.3	16.3
N113 to V104	26.5	26.4	26.4	26.5	21	27.2	27.2	26.3	26.3

## Data Availability

All datasets compiled and analyzed here are provided in the Supplementary Information accompanying this manuscript.

## Supplementary Materials

The following supporting information can be downloaded at https://www.mdpi.com/article/10.3390/biology12111370/s1. Figure S1: Distance measurements of S53-H87-A223; Figure S2: Distance measurements of K81-V104-N113; Figure S3: Per-residue All-atom and Cα B′-factors; Table S1: PDB IDs for Mouse structures analyzed; Table S2: PDB IDs for human structures analyzed; Table S3: Measurement 5: Opening and closing dynamics of the Kinase catalytic domain; Table S4: Measurement 6: Hydrophobic core and N-lobe dynamics; Table S5: PDB ID, Space group, Resolution and molecules in the ASU for the mouse crystal structure dataset; Table S6: PDB ID, Space group, Resolution and molecules in the ASU for the human crystal structure dataset.

## References

[1] Manning G., Whyte D.B., Martinez R., Hunter T., Sudarsanam S. (2002). The Protein Kinase Complement of the Human Genome. Science.

[2] Cicenas J., Zalyte E., Bairoch A., Gaudet P. (2018). Kinases and Cancer. Cancers.

[3] Ahuja L.G. (2018). Protein Tyrosine Phosphatases.

[4] Bhullar K.S., Lagarón N.O., McGowan E.M., Parmar I., Jha A., Hubbard B.P., Rupasinghe H.P.V. (2018). Kinase-targeted cancer therapies: Progress, challenges and future directions. Mol. Cancer.

[5] Roskoski R. (2023). Properties of FDA-approved small molecule protein kinase inhibitors: A 2023 update. Pharmacol. Res..

[6] Taylor S.S., Kornev A.P. (2011). Protein kinases: Evolution of dynamic regulatory proteins. Trends Biochem. Sci..

[7] Taylor S.S., Wu J., Bruystens J.G.H., Del Rio J.C., Lu T.-W., Kornev A.P., Ten Eyck L.F. (2021). From structure to the dynamic regulation of a molecular switch: A journey over 3 decades. J. Biol. Chem..

[8] Taylor S.S., Ilouz R., Zhang P., Kornev A.P. (2012). Assembly of allosteric macromolecular switches: Lessons from PKA. Nat. Rev. Mol. Cell Biol..

[9] Lu T.-W., Aoto P.C., Weng J.-H., Nielsen C., Cash J.N., Hall J., Zhang P., Simon S.M., Cianfrocco M.A., Taylor S.S. (2020). Structural analyses of the PKA RIIβ holoenzyme containing the oncogenic DnaJB1-PKAc fusion protein reveal protomer asymmetry and fusion-induced allosteric perturbations in fibrolamellar hepatocellular carcinoma. PLoS Biol..

[10] Zhang P., Knape M.J., Ahuja L.G., Keshwani M.M., King C.C., Sastri M., Herberg F.W., Taylor S.S. (2015). Single Turnover Autophosphorylation Cycle of the PKA RIIβ Holoenzyme. PLoS Biol..

[11] Wong W., Scott J.D. (2004). AKAP signalling complexes: Focal points in space and time. Nat. Rev. Mol. Cell Biol..

[12] Amer Y.O., Hebert-Chatelain E. (2018). Mitochondrial cAMP-PKA signaling: What do we really know?. Biochim. Biophys. Acta BBA—Bioenerg..

[13] Zheng L., Yu L., Tu Q., Zhang M., He H., Chen W., Gao J., Yu J., Wu Q., Zhao S. (2000). Cloning and mapping of human PKIB and PKIG, and comparison of tissue expression patterns of three members of the protein kinase inhibitor family, including PKIA. Biochem. J..

[14] Grisan F., Iannucci L.F., Surdo N.C., Gerbino A., Zanin S., Di Benedetto G., Pozzan T., Lefkimmiatis K. (2021). PKA compartmentalization links cAMP signaling and autophagy. Cell Death Differ..

[15] Lu T.-W., Wu J., Aoto P.C., Weng J.-H., Ahuja L.G., Sun N., Cheng C.Y., Zhang P., Taylor S.S. (2019). Two PKA RIα holoenzyme states define ATP as an isoform-specific orthosteric inhibitor that competes with the allosteric activator, cAMP. Proc. Natl. Acad. Sci. USA.

[16] Kim C., Cheng C.Y., Saldanha S.A., Taylor S.S. (2007). PKA-I Holoenzyme Structure Reveals a Mechanism for cAMP-Dependent Activation. Cell.

[17] Boettcher A.J., Wu J., Kim C., Yang J., Bruystens J., Cheung N., Pennypacker J.K., Blumenthal D.A., Kornev A.P., Taylor S.S. (2011). Realizing the Allosteric Potential of the Tetrameric Protein Kinase A RIα Holoenzyme. Structure.

[18] Mayr B., Montminy M. (2001). Transcriptional regulation by the phosphorylation-dependent factor CREB. Nat. Rev. Mol. Cell Biol..

[19] Zhang H., Kong Q., Wang J., Jiang Y., Hua H. (2020). Complex roles of cAMP–PKA–CREB signaling in cancer. Exp. Hematol. Oncol..

[20] Yang J., Ten Eyck L.F., Xuong N.-H., Taylor S.S. (2004). Crystal Structure of a cAMP-dependent Protein Kinase Mutant at 1.26 Å: New Insights into the Catalytic Mechanism. J. Mol. Biol..

[21] Akamine P., Madhusudan, Wu J., Xuong N.-H., Eyck L.F., Taylor S.S. (2003). Dynamic Features of cAMP-dependent Protein Kinase Revealed by Apoenzyme Crystal Structure. J. Mol. Biol..

[22] Taylor S.S., Søberg K., Kobori E., Wu J., Pautz S., Herberg F.W., Skålhegg B.S. (2022). The Tails of Protein Kinase A. Mol. Pharmacol..

[23] Meharena H.S., Fan X., Ahuja L.G., Keshwani M.M., McClendon C.L., Chen A.M., Adams J.A., Taylor S.S. (2016). Decoding the Interactions Regulating the Active State Mechanics of Eukaryotic Protein Kinases. PLoS Biol..

[24] Carrera A.C., Alexandrov K., Roberts T.M. (1993). The conserved lysine of the catalytic domain of protein kinases is actively involved in the phosphotransfer reaction and not required for anchoring ATP. Proc. Natl. Acad. Sci. USA.

[25] Masterson L.R., Cembran A., Shi L., Veglia G. (2012). Allostery and Binding Cooperativity of the Catalytic Subunit of Protein Kinase A by NMR Spectroscopy and Molecular Dynamics Simulations. Adv. Protein Chem. Struct. Biol..

[26] Zheng J., Knighton D.R., Xuong N.H., Taylor S.S., Sowadski J.M., Ten Eyck L.F. (1993). Crystal structures of the myristylated catalytic subunit of cAMP-dependent protein kinase reveal open and closed conformations. Protein Sci..

[27] Lauber B.S., Hardegger L.A., Alam K.A., Lund B.A., Dumele O., Harder M., Kuhn B., Engh R.A., Diederich F. (2016). Addressing the Glycine-Rich Loop of Protein Kinases by a Multi-Facetted Interaction Network: Inhibition of PKA and a PKB Mimic. Chem.—Eur. J..

[28] Steinberg S.F. (2018). Post-translational modifications at the ATP-positioning G-loop that regulate protein kinase activity. Pharmacol. Res..

[29] Aimes R.T., Hemmer W., Taylor S.S. (2000). Serine-53 at the Tip of the Glycine-Rich Loop of cAMP-Dependent Protein Kinase: Role in Catalysis, P-Site Specificity, and Interaction with Inhibitors. Biochemistry.

[30] Cui Y., Sun G. (2019). Structural versatility that serves the function of the HRD motif in the catalytic loop of protein tyrosine kinase, Src. Protein Sci..

[31] La Sala G., Riccardi L., Gaspari R., Cavalli A., Hantschel O., De Vivo M. (2016). HRD Motif as the Central Hub of the Signaling Network for Activation Loop Autophosphorylation in Abl Kinase. J. Chem. Theory Comput..

[32] Steichen J.M., Kuchinskas M., Keshwani M.M., Yang J., Adams J.A., Taylor S.S. (2012). Structural Basis for the Regulation of Protein Kinase A by Activation Loop Phosphorylation. J. Biol. Chem..

[33] Keshwani M.M., Klammt C., von Daake S., Ma Y., Kornev A.P., Choe S., Insel P.A., Taylor S.S. (2012). Cotranslational cis-phosphorylation of the COOH-terminal tail is a key priming step in the maturation of cAMP-dependent protein kinase. Proc. Natl. Acad. Sci. USA.

[34] Cox S., Taylor S.S. (1995). Kinetic Analysis of cAMP-Dependent Protein Kinase: Mutations at Histidine 87 Affect Peptide Binding and pH Dependence. Biochemistry.

[35] Humphries K.M., Deal M.S., Taylor S.S. (2005). Enhanced dephosphorylation of cAMP-dependent protein kinase by oxidation and thiol modification. J. Biol. Chem..

[36] Kornev A.P., Haste N.M., Taylor S.S., Ten Eyck L.F. (2006). Surface comparison of active and inactive protein kinases identifies a conserved activation mechanism. Proc. Natl. Acad. Sci. USA.

[37] Hu J., Ahuja L.G., Meharena H.S., Kannan N., Kornev A.P., Taylor S.S., Shaw A.S. (2015). Kinase Regulation by Hydrophobic Spine Assembly in Cancer. Mol. Cell. Biol..

[38] Ahuja L.G., Kornev A.P., McClendon C.L., Veglia G., Taylor S.S. (2017). Mutation of a kinase allosteric node uncouples dynamics linked to phosphotransfer. Proc. Natl. Acad. Sci. USA.

[39] Ahuja L.G., Aoto P.C., Kornev A.P., Veglia G., Taylor S.S. (2019). Dynamic allostery-based molecular workings of kinase:peptide complexes. Proc. Natl. Acad. Sci. USA.

[40] Wang Y., Manu V.S., Kim J., Li G., Ahuja L.G., Aoto P., Taylor S.S., Veglia G. (2019). Globally correlated conformational entropy underlies positive and negative cooperativity in a kinase’s enzymatic cycle. Nat. Commun..

[41] Khavrutskii I.V., Grant B., Taylor S.S., McCammon J.A. (2009). A Transition Path Ensemble Study Reveals a Linchpin Role for Mg^2+^ during Rate-Limiting ADP Release from Protein Kinase A. Biochemistry.

[42] Armstrong R.N., Kondo H., Granot J., Kaiser E.T., Mildvan A.S. (1979). Magnetic resonance and kinetic studies of the manganese(II) ion and substrate complexes of the catalytic subunit of adenosine 3’,5’-monophosphate dependent protein kinase from bovine heart. Biochemistry.

[43] Cheng Y., Zhang Y., McCammon J.A. (2005). How Does the cAMP-Dependent Protein Kinase Catalyze the Phosphorylation Reaction: An ab Initio QM/MM Study. J. Am. Chem. Soc..

[44] Hart J.C., Sheppard D.W., Hillier I.H., Burton N.A. (1999). What is the mechanism of phosphoryl transfer in protein kinases? A hybrid quantum mechanical/molecular mechanical study. Chem. Commun..

[45] Sheppard D., Burton N., Hillier I. (2000). Ab initio hybrid quantum mechanical/molecular mechanical studies of the mechanisms of the enzymes protein kinase and thymidine phosphorylase. J. Mol. Struct. Theochem..

[46] Valiev M., Kawai R., Adams J.A., Weare J.H. (2003). The Role of the Putative Catalytic Base in the Phosphoryl Transfer Reaction in a Protein Kinase: First-Principles Calculations. J. Am. Chem. Soc..

[47] Díaz N., Field M.J. (2004). Insights into the Phosphoryl-Transfer Mechanism of cAMP-Dependent Protein Kinase from Quantum Chemical Calculations and Molecular Dynamics Simulations. J. Am. Chem. Soc..

[48] Henkelman G., LaBute M.X., Tung C.-S., Fenimore P.W., McMahon B.H. (2005). Conformational dependence of a protein kinase phosphate transfer reaction. Proc. Natl. Acad. Sci. USA.

[49] Valiev M., Yang J., Adams J.A., Taylor S.S., Weare J.H. (2007). Phosphorylation Reaction in cAPK Protein Kinase-Free Energy Quantum Mechanical/Molecular Mechanics Simulations. J. Phys. Chem. B.

[50] Montenegro M., Garcia-Viloca M., Lluch J.M., González-Lafont A. (2011). A QM/MM study of the phosphoryl transfer to the Kemptide substrate catalyzed by protein kinase A. The effect of the phosphorylation state of the protein on the mechanism. Phys. Chem. Chem. Phys..

[51] Adams J.A. (2001). Kinetic and Catalytic Mechanisms of Protein Kinases. Chem. Rev..

[52] Arter C., Trask L., Ward S., Yeoh S., Bayliss R. (2022). Structural features of the protein kinase domain and targeted binding by small-molecule inhibitors. J. Biol. Chem..

[53] Vijayan R.S.K., He P., Modi V., Duong-Ly K.C., Ma H., Peterson J.R., Dunbrack R.L., Levy R.M. (2015). Conformational Analysis of the DFG-Out Kinase Motif and Biochemical Profiling of Structurally Validated Type II Inhibitors. J. Med. Chem..

[54] Modi V., Dunbrack R.L. (2019). Defining a new nomenclature for the structures of active and inactive kinases. Proc. Natl. Acad. Sci. USA.

[55] Berman H.M., Westbrook J., Feng Z., Gilliland G., Bhat T.N., Weissig H., Shindyalov I.N., Bourne P.E. (2000). The Protein Data Bank. Nucleic Acids Res..

[56] (2015). The PyMOL Molecular Graphics System.

[57] Hunter J.D. (2007). Matplotlib: A 2D graphics environment. Comput. Sci. Eng..

[58] Waskom M.L. (2021). seaborn: Statistical data visualization. J. Open Source Softw..

[59] Smith D.K., Radivojac P., Obradovic Z., Dunker A.K., Zhu G. (2003). Improved amino acid flexibility parameters. Protein Sci..

[60] Barthels F., Schirmeister T., Kersten C. (2021). BANΔIT: B’-Factor Analysis for Drug Design and Structural Biology. Mol. Inform..

[61] Cock P.J.A., Antao T., Chang J.T., Chapman B.A., Cox C.J., Dalke A., Friedberg I., Hamelryck T., Kauff F., Wilczynski B. (2009). Biopython: Freely available Python tools for computational molecular biology and bioinformatics. Bioinformatics.

[62] Pedregosa F., Varoquaux G., Gramfort A., Michel V., Thirion B., Grisel O., Blondel M., Prettenhofer P., Weiss R., Dubourg V. (2011). Scikit-learn: Machine Learning in Python. J. Mach. Learn. Res..

[63] Yang J., Wu J., Steichen J.M., Kornev A.P., Deal M.S., Li S., Sankaran B., Woods V.L., Taylor S.S. (2012). A Conserved Glu–Arg Salt Bridge Connects Coevolved Motifs That Define the Eukaryotic Protein Kinase Fold. J. Mol. Biol..

[64] Wu J., Yang J., Kannan N., Madhusudan, Xuong N.-H., Ten Eyck L.F., Taylor S.S. (2005). Crystal structure of the E230Q mutant of cAMP-dependent protein kinase reveals an unexpected apoenzyme conformation and an extended N-terminal A helix. Protein Sci..

[65] Yang J., Kennedy E.J., Wu J., Deal M.S., Pennypacker J., Ghosh G., Taylor S.S. (2009). Contribution of Non-catalytic Core Residues to Activity and Regulation in Protein Kinase A. J. Biol. Chem..

[66] Möbitz H. (2015). The ABC of protein kinase conformations. Biochim. Biophys. Acta BBA—Proteins Proteom..

[67] Ung P.M.-U., Rahman R., Schlessinger A. (2018). Redefining the Protein Kinase Conformational Space with Machine Learning. Cell Chem. Biol..

[68] Jacobs M.D., Caron P.R., Hare B.J. (2007). Classifying protein kinase structures guides use of ligand-selectivity profiles to predict inactive conformations: Structure of lck/imatinib complex. Proteins Struct. Funct. Bioinform..

[69] Brooijmans N., Chang Y.-W., Mobilio D., Denny R.A., Humblet C. (2010). An enriched structural kinase database to enable kinome-wide structure-based analyses and drug discovery. Protein Sci..

[70] Sun Z., Liu Q., Qu G., Feng Y., Reetz M.T. (2019). Utility of B-Factors in Protein Science: Interpreting Rigidity, Flexibility, and Internal Motion and Engineering Thermostability. Chem. Rev..

