# Supplementary material for: Crystal Structures Reveal Hidden Domain Mechanics in Protein Kinase A (PKA)
Source: Biology (Basel). 2023 Oct 26;12(11):1370. doi: 10.3390/biology12111370 (PMC10669547; doi:10.3390/biology12111370)
Supplement: Supplementary file 1 [file biology-12-01370-s001.zip › biology-2633674-supplementary.pdf]

Supporting Information for

Crystal structures reveal hidden domain mechanics in Protein Kinase A (PKA)

Colin L. Welsh[1,*], Abigail E. Conklin[1,*], Lalima K. Madan[1,2,†]

[1]Department of Cellular and Molecular Pharmacology and Experimental Therapeutics, College of Medicine, Medical University of South Carolina; [2]Hollings Cancer Center, Medical University of South Carolina

[*]Authors contributed equally

[†]Corresponding Author. All queries may be emailed to LKM at madanl@musc.edu

Mailing Address:

173 Ashley Avenue, MSC 509

Basic Sciences Building Rm 358

Medical University of South Carolina

Charleston, SC-29425

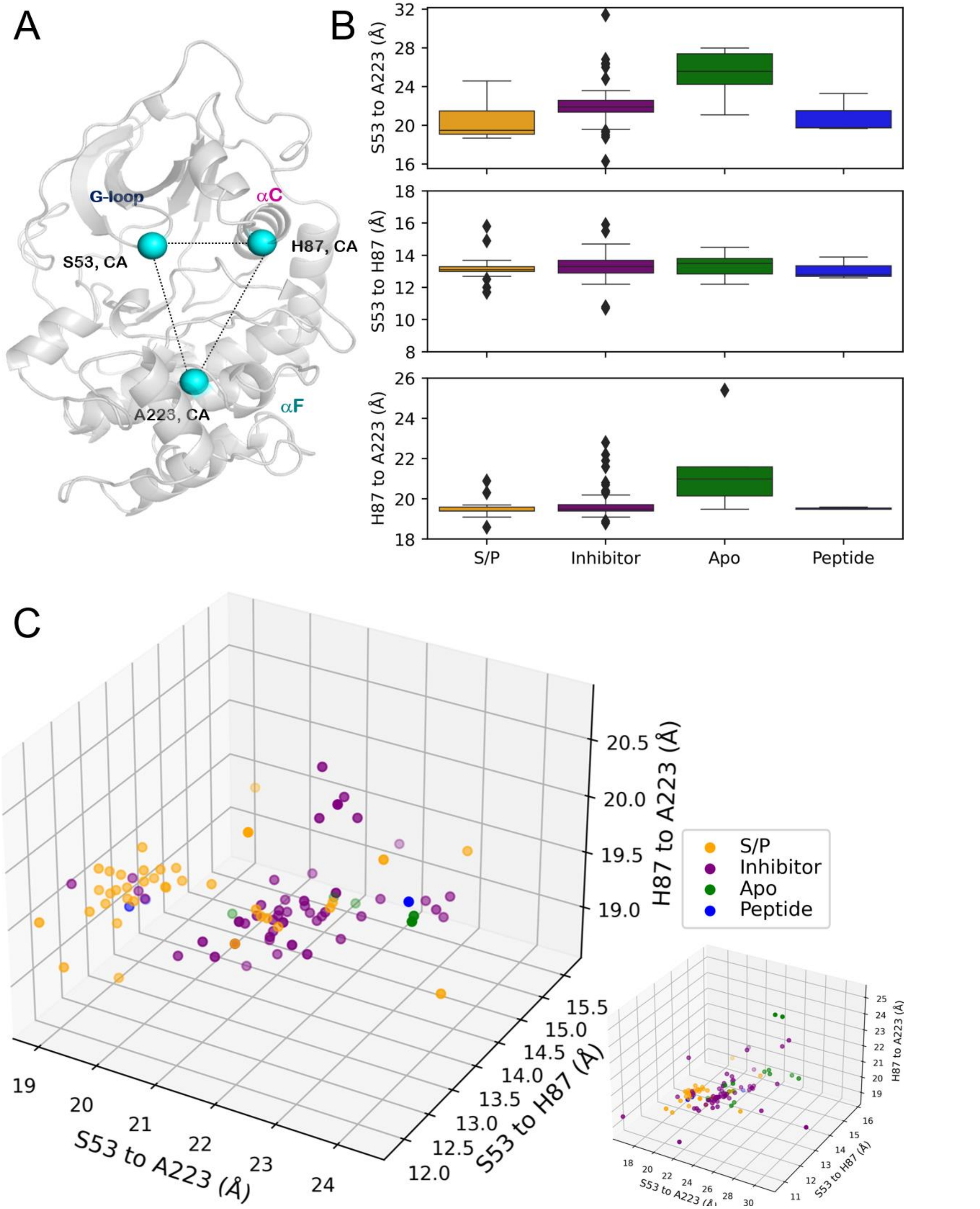

Supplemental Figure S1: (A) Analyzed distances on the structure of PKA (PDB ID: 1ATP). **(B)** Box-and-whisker plots of C_α-C_α distances. Top, S53-A223. Middle, S53-H87. Bottom, H87-A223. **(C)** 3D scatterplot of distances. Left, zoomed plot showing main clustering of points. Bottom right, full plot including outliers.

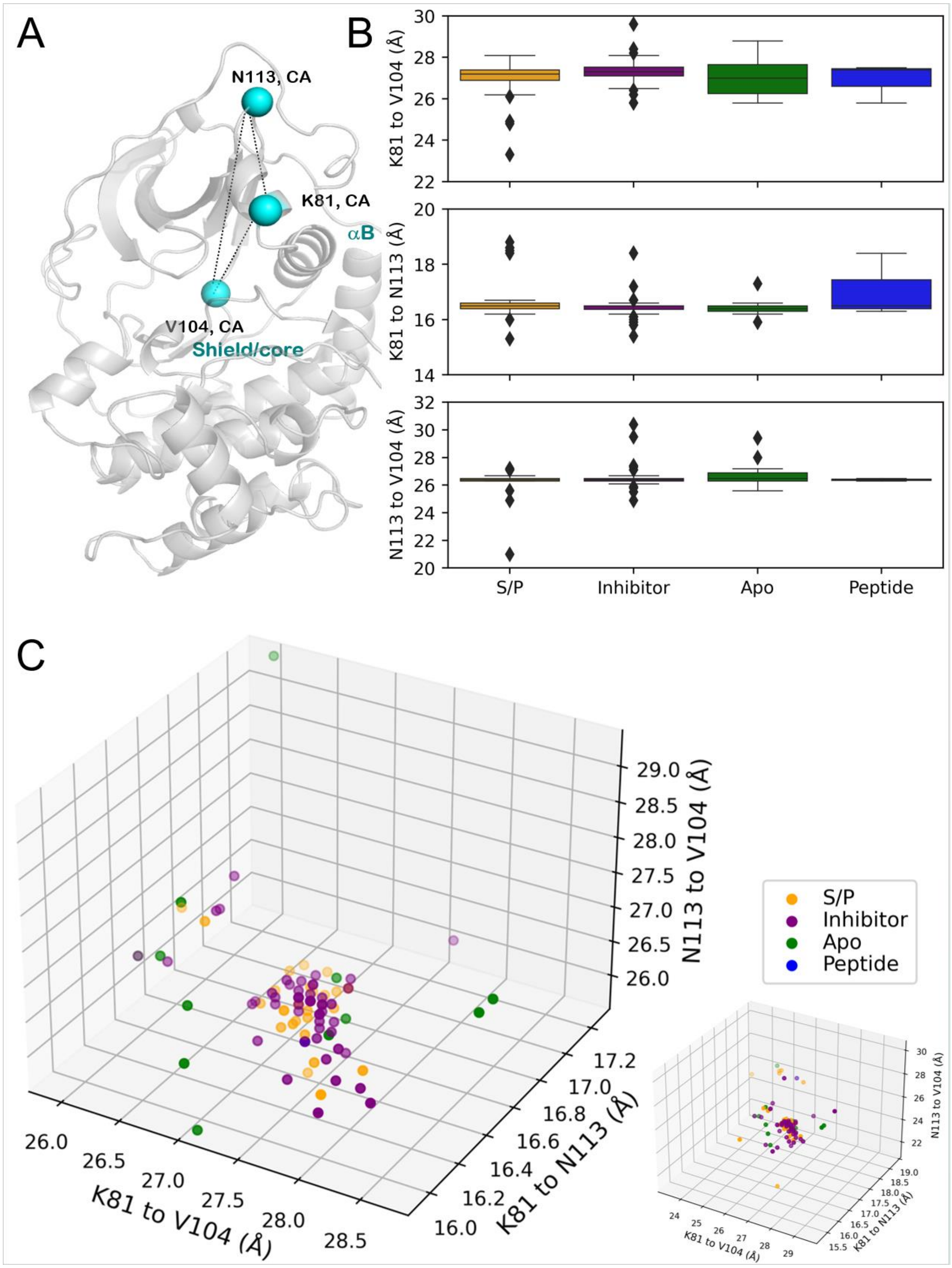

Supplemental Figure S2: (A) Analyzed distances on the structure of PKA (PDB ID: 1ATP). **(B)** Box-and-whisker plots of C_{α}-C_{α} distances. Top, K81-V104. Middle, K81-N113. Bottom, N113-V104. **(C)** 3D scatterplot of distances. Left, zoomed plot showing main clustering of points. Bottom right, full plot including outliers.

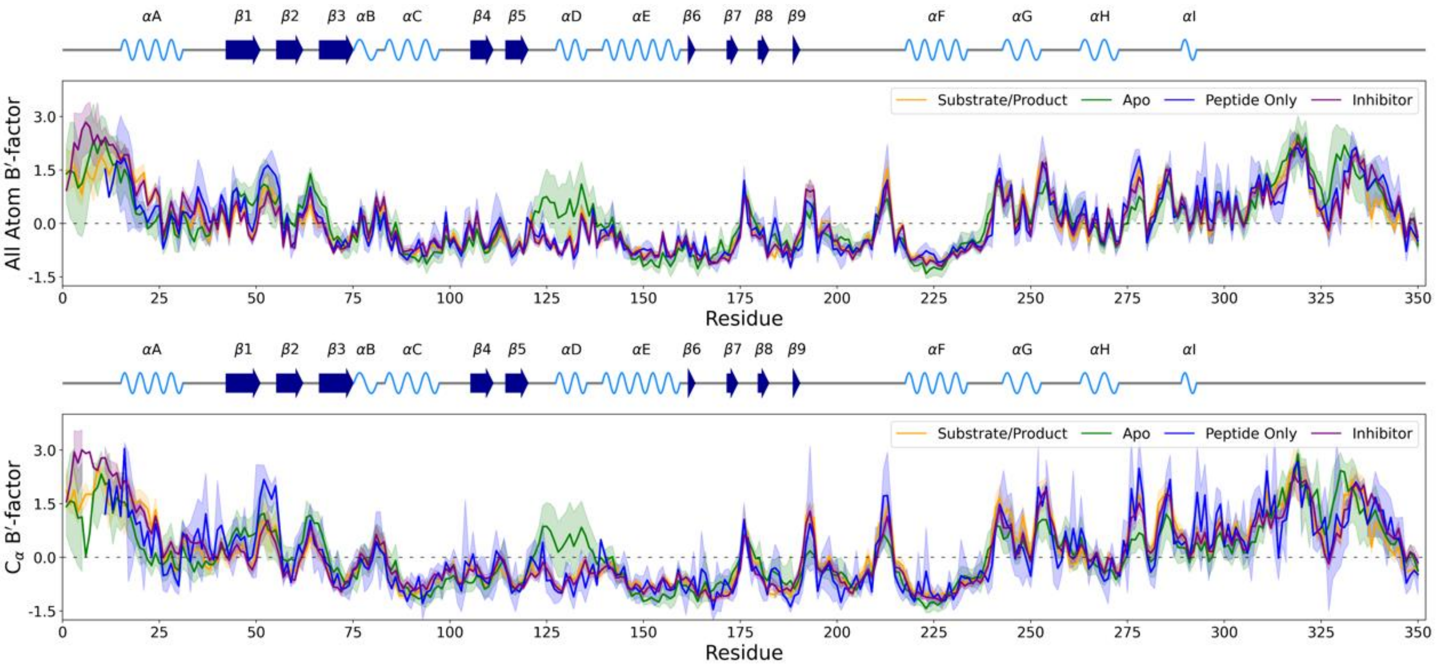

Supplemental Figure S3: Per-residue B′-factors from all heavy atoms (top) and C_α atoms only (bottom).

Table S1: Mouse structures analyzed. (N=65). Also see Table S5.

Apo	Substrate/Product	Inhibitor	Peptide only
4NTS_A	7V0G	7E12	4O22
4NTS_B	7UJX	7E11	2CPK
4DG2	3X2W	7E0Z	1APM
4DFZ	3X2V	4XW4	
1JLU	3X2U	4IAC	
1J3H_A	4XW6	4HPU	
1J3H_B	4XW5	4HPT	
4DFY_A	4NTT_A	4DH8	
4DFY_B	4NTT_B	4DH7	
1SYK_A	4O21	4DG0	
1SYK_B	4IB3	4DFX	
	4IB1	3OW3	
	4IB0	2ERZ	
	4IAZ	1REK	
	4IAY	1REJ	
	4IAK	1RE8	
	4IAI	1BX6	
	4IAF	1BKX	
	4IAD	1FMO	
	6E21	4DG3	
	4DH5		
	4DH1		
	3FJQ		
	1L3R		
	1JBP		
	1ATP		
	4DH3		
	3QAM		
	3QAL		
	2QUR		
	1RDQ		

Table S2: Human structures analyzed. (N=50). Also see Table S6.

Apo	Substrate/Product	Inhibitor
4AE9_A	4WB8	7Y1G_A
4AE9_B	4WB7_A	7Y1G_B
4AE6_A	4WB7_B	6QJ7
4AE6_B	4WB6_A	6FRX
	4WB6_B	6C0U
	4WB5	5N23
		5UZK
		5BX7
		5BX6
		5J5X
		5IZJ_A
		5IZJ_B
		5IZF
		4UJB
		4UJA
		4UJ9
		4UJ2
		4UJ1
		3VQH
		3POO
		3P0M
		3OXT
		3OWP
		3OVV
		3OOG
		3AMB
		3AMA
		3NX8
		3L9N
		3L9M_A
		3L9M_B
		3L9L_A
		3L9L_B
		3AGM
		3AGL_A
		3AGL_B
		3MVJ_A
		3MVJ_B
		3MVJ_C
		2GU8

Table S3: Measurement 5: Opening and closing dynamics of the Kinase catalytic domain

Distance (Å)	S53(Cα) to A223(Cα)	S53(Cα) to H87(Cα)	H87(Cα) to A223(Cα)
Substrate/Product			
Average	20.21	13.15	19.59
Median	19.50	13.10	19.50
Std Dev	1.58	0.70	0.61
N	37.00	37.00	37.00
Min Value	18.70	11.70	19.10
1st Quartile	19.10	13.00	19.40
2nd Quartile	19.50	13.10	19.50
3rd Quartile	21.50	13.30	19.60
Max Value	24.60	15.80	22.70
Inhibitor			
Average	22.17	13.35	19.76
Median	21.90	13.30	19.50
Std Dev	2.16	0.89	0.78
N	58.00	58.00	58.00
Min Value	16.30	10.70	18.60
1st Quartile	21.33	12.90	19.40
2nd Quartile	21.90	13.30	19.50
3rd Quartile	22.60	13.68	19.78
Max Value	31.40	15.90	22.80
Apo			
Average	25.37	13.42	20.83
Median	25.60	13.50	20.70
Std Dev	2.25	0.74	1.61
N	15.00	15.00	15.00
Min Value	21.10	12.20	18.20
1st Quartile	24.25	12.85	19.85
2nd Quartile	25.60	13.50	20.70
3rd Quartile	27.40	13.80	21.55
Max Value	28.00	14.50	25.40
Peptide			
Average	20.93	13.10	19.53
Median	19.80	12.80	19.50
Std Dev	2.05	0.70	0.06
N	3.00	3.00	3.00
Min Value	19.70	12.60	19.50
1st Quartile	19.75	12.70	19.50
2nd Quartile	19.80	12.80	19.50
3rd Quartile	21.55	13.35	19.55
Max Value	23.30	13.90	19.60

Table S4: Measurement 6: Hydrophobic core and N-lobe dynamics

Distance (Å)	K81(Cα) to V104(Cα)	K81(Cα) to N113(Cα)	N113(Cα) to V104(Cα)
Substrate/Product			
Average	26.89	16.71	26.25
Median	27.20	16.50	26.40
Std Dev	0.98	0.79	0.96
N	37.00	37.00	37.00
Min Value	23.30	15.30	21.00
1st Quartile	26.90	16.40	26.30
2nd Quartile	27.20	16.50	26.40
3rd Quartile	27.40	16.60	26.50
Max Value	28.10	18.80	27.20
Inhibitor			
Average	27.35	16.41	26.50
Median	27.35	16.50	26.40
Std Dev	0.55	0.27	0.74
N	58.00	58.00	58.00
Min Value	25.80	15.40	24.90
1st Quartile	27.13	16.40	26.30
2nd Quartile	27.35	16.50	26.40
3rd Quartile	27.58	16.50	26.50
Max Value	29.60	17.20	30.40
Apo			
Average	27.03	16.40	26.77
Median	27.00	16.40	26.50
Std Dev	0.97	0.33	0.91
N	15.00	15.00	15.00
Min Value	25.80	15.90	25.60
1st Quartile	26.25	16.30	26.30
2nd Quartile	27.00	16.40	26.50
3rd Quartile	27.65	16.50	26.90
Max Value	28.80	17.30	29.40
Peptide			
Average	26.90	17.07	26.40
Median	27.40	16.50	26.40
Std Dev	0.95	1.16	0.10
N	3.00	3.00	3.00
Min Value	25.80	16.30	26.30
1st Quartile	26.60	16.40	26.35
2nd Quartile	27.40	16.50	26.40
3rd Quartile	27.45	17.45	26.45
Max Value	27.50	18.40	26.50

Table S5: PDB ID, Space group, Resolution and molecules in the ASU for the mouse crystal structure dataset.

Entry ID	Chains	Resolution (Å)	Space Group	Chain ID	Macromolecule Name
7V0G	2	1.63	P 21 21 21	A	cAMP-dependent protein kinase catalytic subunit alpha
				B	Peptide from cAMP-dependent protein kinase inhibitor alpha
7UJX	2	2.4	P 21 21 21	A	cAMP-dependent protein kinase catalytic subunit alpha
				B	Peptide from cAMP-dependent protein kinase inhibitor alpha
3X2W	2	1.7	P 21 21 21	A	cAMP-dependent protein kinase catalytic subunit alpha
				B	Peptide from cAMP-dependent protein kinase inhibitor alpha
3X2V	2	1.77	P 21 21 21	A	cAMP-dependent protein kinase catalytic subunit alpha
				B	Substrate Peptide
3X2U	2	2.4	P 21 21 21	A	cAMP-dependent protein kinase catalytic subunit alpha
				B	Substrate Peptide
4XW6	2	1.9	P 21 21 21	A	cAMP-dependent protein kinase catalytic subunit alpha
				B	Peptide from cAMP-dependent protein kinase inhibitor alpha
4XW5	2	1.95	P 21 21 21	A	cAMP-dependent protein kinase catalytic subunit alpha
				B	Peptide from cAMP-dependent protein kinase inhibitor alpha
4NTT	2	3.5	P 21 21 21	A	cAMP-dependent protein kinase catalytic subunit alpha
				B	
4O21	2	1.95	P 21 21 21	A	cAMP-dependent protein kinase catalytic subunit alpha
				B	Thio-phosphorylated peptide pSP20
4IB3	2	2.2	P 21 21 21	A	cAMP-dependent protein kinase catalytic subunit alpha
				B	phosphorylated pseudo-substrate peptide pSP20
4IB1	2	1.63	P 21 21 21	A	cAMP-dependent protein kinase catalytic subunit alpha
				B	phosphorylated pseudo-substrate peptide pSP20
4IB0	2	1.87	P 21 21 21	A	cAMP-dependent protein kinase catalytic subunit alpha
				B	phosphorylated pseudo-substrate peptide pSP20
4IAZ	2	1.85	P 21 21 21	A	cAMP-dependent protein kinase catalytic subunit alpha
				B	phosphorylated pseudo-substrate peptide pSP20
4IAY	2	2	P 21 21 21	A	cAMP-dependent protein kinase catalytic subunit alpha
				B	phosphorylated pseudo-substrate peptide pSP20
4IAK	2	1.6	P 21 21 21	A	cAMP-dependent protein kinase catalytic subunit alpha
				B	phosphorylated pseudo-substrate peptide pSP20
4IAI	2	1.55	P 21 21 21	A	cAMP-dependent protein kinase catalytic subunit alpha
				B	phosphorylated pseudo-substrate peptide pSP20
4IAF	2	2.2	P 21 21 21	A	cAMP-dependent protein kinase catalytic subunit alpha
				B	phosphorylated pseudo-substrate peptide pSP20
4IAD	2	1.9	P 21 21 21	A	cAMP-dependent protein kinase catalytic subunit alpha
				B	Phosphorylated peptide pSP20
6E 21	2	2.5	P 21 21 21	A	cAMP-dependent protein kinase catalytic subunit alpha
				B	pSP20
4DH5	2	2.2	P 21 21 21	A	cAMP-dependent protein kinase catalytic subunit alpha
				B	cAMP-dependent protein kinase inhibitor alpha
4DH3	2	2.2	P 21 21 21	A	cAMP-dependent protein kinase catalytic subunit alpha

				B	cAMP-dependent protein kinase inhibitor alpha
4DH1	2	2	P 21 21 21	A	cAMP-dependent protein kinase catalytic subunit alpha
				B	cAMP-dependent protein kinase inhibitor alpha
3QA M	2	1.92	P 21 21 21	A	cAMP-dependent protein kinase catalytic subunit alpha
				B	Protein kinase inhibitor
3QAL	2	1.7	P 21 21 21	A	cAMP-dependent protein kinase catalytic subunit alpha
				B	Protein kinase inhibitor
3FJQ	2	1.6	P 21 21 21	A	cAMP-dependent protein kinase catalytic subunit alpha
				B	cAMP-dependent protein kinase inhibitor alpha
2QUR	2	2.5	P 21 21 21	A	cAMP-dependent protein kinase, alpha-catalytic subunit
				B	20-mer fragment from cAMP-dependent protein kinase inhibitor alpha
1RDQ	2	1.26	P 21 21 21	A	cAMP-dependent protein kinase, alpha-catalytic subunit
				B	cAMP-dependent protein kinase inhibitor, alpha form
1L3R	2	2	P 21 21 21	A	CAMP-DEPENDENT PROTEIN KINASE, ALPHA-CATALYTIC SUBUNIT
				B	CAMP-DEPENDENT PROTEIN KINASE INHIBITOR, MUSCLE/BRAIN FORM
1JBP	2	2.2	P 21 21 21	A	CAMP-DEPENDENT PROTEIN KINASE, ALPHA-CATALYTIC SUBUNIT
				B	CAMP-DEPENDENT PROTEIN KINASE INHIBITOR, MUSCLE/BRAIN FORM
1ATP	2	2.2	P 21 21 21	A	cAMP-DEPENDENT PROTEIN KINASE
				B	PEPTIDE INHIBITOR PKI(5-24)
4NTS	2	2.9	P 21 21 21	A	cAMP-dependent protein kinase catalytic subunit alpha
				B	
4DG2	2	2	P 21 21 21	A	cAMP-dependent protein kinase catalytic subunit alpha
				B	cAMP-dependent protein kinase inhibitor alpha
4DFZ	2	2	P 21 21 21	A	cAMP-dependent protein kinase catalytic subunit alpha
				B	cAMP-dependent protein kinase inhibitor alpha
1JLU	2	2.25	P 21 21 21	A	AMP-DEPENDENT PROTEIN KINASE, ALPHA-CATALYTIC SUBUNIT
				B	CAMP-DEPENDENT PROTEIN KINASE INHIBITOR, MUSCLE/BRAIN FORM
1J3H	2	2.9	P 21 21 21	A	cAMP-dependent protein kinase, alpha-catalytic subunit
				B	
4DFY	2	2.997	P 21 21 21	A	cAMP-dependent protein kinase catalytic subunit alpha
				B	
1SYK	2	2.8	P 21 21 21	A	cAMP-dependent protein kinase, alpha-catalytic subunit
				B	
1APM	2	2	P 21 21 21	A	cAMP-DEPENDENT PROTEIN KINASE
				B	PEPTIDE INHIBITOR PKI(5-24)
4O22	2	1.7	P 21 21 21	A	cAMP-dependent protein kinase catalytic subunit alpha.
				B	Phosphorylated peptide pSP20.
2CPK	2	2.7	P 21 21 21	A	cAMP-DEPENDENT PROTEIN KINASE, CATALYTIC SUBUNIT
				B	PEPTIDE INHIBITOR 20-MER
7E 12	2	2.796	P 21 21 21	A	THR-ARG-SER-GLU-ILE-ARG-ARG-ALA-SER-THR-ILE-GLU
				B	cAMP-dependent protein kinase catalytic subunit alpha

7E 11	2	3.43	P 21 21 21	A	cAMP-dependent protein kinase catalytic subunit alpha
				B	PLN
7E0Z	2	2.162	P 21 21 21	A	cAMP-dependent protein kinase catalytic subunit alpha
				B	PLN
4XW4	2	1.82	P 21 21 21	A	cAMP-dependent protein kinase catalytic subunit alpha
				B	cAMP-dependent protein kinase inhibitor alpha
4IAC	2	2.15	P 21 21 21	A	cAMP-dependent protein kinase catalytic subunit alpha
				B	Peptide SP20
4HPU	2	1.55	P 21 21 21	A	cAMP-dependent protein kinase catalytic subunit alpha
				B	cAMP-dependent protein kinase inhibitor alpha
4HPT	2	2.15	P 21 21 21	A	cAMP-dependent protein kinase catalytic subunit alpha
				B	cAMP-dependent protein kinase inhibitor alpha
4DG3	2	1.8	P 21 21 21	A	cAMP-dependent protein kinase catalytic subunit alpha
				B	cAMP-dependent protein kinase inhibitor alpha
4DH8	2	2.3	P 21 21 21	A	cAMP-dependent protein kinase catalytic subunit alpha
				B	cAMP-dependent protein kinase inhibitor alpha
4DH7	2	1.8	P 21 21 21	A	cAMP-dependent protein kinase catalytic subunit alpha
				B	cAMP-dependent protein kinase inhibitor alpha
4DG0	2	2	P 21 21 21	A	cAMP-dependent protein kinase catalytic subunit alpha
				B	cAMP-dependent protein kinase inhibitor alpha
4DFX	2	1.35	P 21 21 21	A	cAMP-dependent protein kinase catalytic subunit alpha
				B	cAMP-dependent protein kinase inhibitor alpha
3OW3	2	1.9	P 21 21 21	A	cAMP-dependent protein kinase catalytic subunit alpha
				B	cAMP-dependent protein kinase inhibitor alpha
2ERZ	2	2.2	P 21 21 21	A	cAMP-dependent protein kinase catalytic subunit alpha
				B	cAMP-dependent protein kinase inhibitor alpha
1REK	1	2.3	P 21 21 21	A	cAMP-dependent protein kinase, alpha-catalytic subunit
1REJ	1	2.2	P 21 21 21	A	cAMP-dependent protein kinase, alpha-catalytic subunit
1RE8	1	2.1	P 21 21 21	A	cAMP-dependent protein kinase, alpha-catalytic subunit
1BX6	1	2.1	P 21 21 21	A	CAMP-DEPENDENT PROTEIN KINASE
1BKX	2	2.6	P 21 21 21	A	CAMP-DEPENDENT PROTEIN KINASE
1FMO	2	2.2	P 21 21 21	A	CAMP-DEPENDENT PROTEIN KINASE
				B	HEAT STABLE RABBIT SKELETAL MUSCLE INHIBITOR PROTEIN
4NTS	2	2.9	P 21 21 21	A	cAMP-dependent protein kinase catalytic subunit alpha
				B	
4NTT	2	3.5	P 21 21 21	A	cAMP-dependent protein kinase catalytic subunit alpha
				B	
1J3H	2	2.9	P 21 21 21	A	cAMP-dependent protein kinase, alpha-catalytic subunit
				B	
1SYK	2	2.8	P 21 21 21	A	cAMP-dependent protein kinase, alpha-catalytic subunit
				B	
4DFY	2	2.997	P 21 21 21	A	cAMP-dependent protein kinase, alpha-catalytic subunit
				B	

Table S6: PDB ID, Space group, Resolution and molecules in the ASU for the human crystal structure dataset.

Entry ID	Chains	Resolution (Å)	Space Group	Chain ID	Macromolecule Name
7Y1G	2	2.3	C 1 2 1	A	cAMP-dependent protein kinase catalytic subunit alpha
				B	
6QJ7	2	1.69	P 21 21 21	A	cAMP-dependent protein kinase catalytic subunit alpha
				B	cAMP-dependent protein kinase inhibitor alpha
6FRX	2	1.88	P 21 21 21	A	cAMP-dependent protein kinase catalytic subunit alpha
				B	cAMP-dependent protein kinase inhibitor alpha
6C0U	1	2.65	P 31 2 1	A	cAMP-dependent protein kinase catalytic subunit alpha
5N23	2	2.088	P 21 21 21	A	cAMP-dependent protein kinase catalytic subunit alpha
				B	cAMP-dependent protein kinase inhibitor alpha
5UZK	2	2.3	P 21 21 21	A	cAMP-dependent protein kinase catalytic subunit alpha
				B	cAMP-dependent protein kinase inhibitor alpha
5BX7	2	1.89	P 21 21 21	A	cAMP-dependent protein kinase catalytic subunit alpha
				B	cAMP-dependent protein kinase inhibitor alpha
5BX6	2	1.89	P 21 21 21	A	cAMP-dependent protein kinase catalytic subunit alpha
				B	cAMP-dependent protein kinase inhibitor alpha
5J5X	2	2.6	P 65 2 2	A	cAMP-dependent protein kinase catalytic subunit alpha
				B	47P-AZ1-DAL-DAR-DAR-DAR-DAR
5IZJ	4	1.85	P 1 21 1	A	cAMP-dependent protein kinase catalytic subunit alpha
				B	
				C	47P-AZ1-DAR-DAR
				D	47P-AZ1-DAR-DAR-DAR
5IZF	2	2.1	P 65 2 2	A	cAMP-dependent protein kinase catalytic subunit alpha
				B	6J9-ZEU-DAR-ACA-DAR-NH2
4UJB	2	1.949	P 21 21 21	A	CAMP-DEPENDENT PROTEIN KINASE CATALYTIC SUBUNIT ALPHA
				B	PEPTIDE
4UJA	2	1.93	P 21 21 21	A	CAMP-DEPENDENT PROTEIN KINASE CATALYTIC SUBUNIT ALPHA
				B	PEPTIDE
4UJ9	2	1.87	P 21 21 21	A	cAMP-dependent protein kinase catalytic subunit alpha
				B	cAMP-dependent protein kinase inhibitor alpha
4UJ2	2	2.019	P 21 21 21	A	cAMP-dependent protein kinase catalytic subunit alpha
				B	cAMP-dependent protein kinase inhibitor alpha
4UJ1	2	1.768	P 21 21 21	A	cAMP-dependent protein kinase catalytic subunit alpha
				B	cAMP-dependent protein kinase inhibitor alpha
3VQH	2	1.95	P 21 21 21	A	cAMP-dependent protein kinase catalytic subunit alpha
				B	cAMP-dependent protein kinase inhibitor alpha
3POO	2	1.6	P 21 21 21	A	cAMP-dependent protein kinase catalytic subunit alpha
				B	cAMP-dependent protein kinase inhibitor alpha
3P0M	2	2.03	P 21 21 21	A	cAMP-dependent protein kinase catalytic subunit alpha
				B	cAMP-dependent protein kinase inhibitor alpha
3OXT	2	2.2	P 21 21 21	A	cAMP-dependent protein kinase catalytic subunit alpha
				B	cAMP-dependent protein kinase inhibitor alpha
3OWP	2	1.88	P 21 21 21	A	cAMP-dependent protein kinase catalytic subunit alpha
				B	cAMP-dependent protein kinase inhibitor alpha

3OVV	2	1.58	P 21 21 21	A	cAMP-dependent protein kinase catalytic subunit alpha
				B	cAMP-dependent protein kinase inhibitor alpha
3OOG	2	2	P 21 21 21	A	cAMP-dependent protein kinase catalytic subunit alpha
				B	cAMP-dependent protein kinase inhibitor alpha
3AMB	2	2.25	P 21 21 21	A	cAMP-dependent protein kinase catalytic subunit alpha
				B	cAMP-dependent protein kinase inhibitor alpha
3AMA	2	1.75	P 21 21 21	A	cAMP-dependent protein kinase catalytic subunit alpha
				B	cAMP-dependent protein kinase inhibitor alpha
3NX8	2	2	P 21 21 21	A	cAMP-dependent protein kinase catalytic subunit alpha
				B	cAMP-dependent protein kinase inhibitor alpha
3L9N	2	2	C 2 2 21	A	cAMP-dependent protein kinase catalytic subunit alpha
				B	cAMP-dependent protein kinase inhibitor alpha
3L9M	4	1.9	C 1 2 1	C	cAMP-dependent protein kinase catalytic subunit alpha
				A	
				B	cAMP-dependent protein kinase inhibitor alpha
				D	
3L9L	4	2	C 1 2 1	C	cAMP-dependent protein kinase catalytic subunit alpha
				A	
				D	cAMP-dependent protein kinase inhibitor alpha
				B	
3AGM	2	2	P 43 21 2	A	cAMP-dependent protein kinase catalytic subunit alpha
				B	N~2~-{8-OXO-8-[4-(9H-PURIN-6-YL)PIPERAZIN-1-YL]OCTANOYL}-D-ARGINYL-D-ARGINYL-D-ARGINYL-D-ARGINYL-D-ARGINYL-D-ARGININAMIDE
3AGL	2	2.1	P 21 21 21	A	cAMP-dependent protein kinase catalytic subunit alpha
				B	
3MVJ	6	2.49	P 21 21 21	A	cAMP-dependent protein kinase catalytic subunit alpha
				B	
				C	
				D	cAMP-dependent protein kinase inhibitor alpha
				E	
				F	
2GU8	2	2.2	P 21 21 21	A	CAMP-dependent protein kinase, alpha-catalytic subunit
				B	inhibitor of CAMP-dependent protein kinase
4WB8	2	1.55	P 21 21 21	A	cAMP-dependent protein kinase catalytic subunit alpha
				B	PKI (5-24)
4WB7	4	1.9	P 21 21 21	A	DnaJ homolog subfamily B member 1,cAMP-dependent protein kinase catalytic subunit alpha
				B	
				C	PKI (5-24)
				D	
4WB6	4	2.1	P 1 21 1	A	cAMP-dependent protein kinase catalytic subunit alpha
				B	cAMP-dependent protein kinase catalytic subunit alpha
				C	PKI (5-24)
				D	
4WB5	2	1.641	P 21 21 21	A	cAMP-dependent protein kinase catalytic subunit alpha
				B	PKI (5-24)

4AE9	2	2.3	P 21 21 21	A	CAMP-DEPENDENT PROTEIN KINASE CATALYTIC SUBUNIT ALPHA
				B	
4AE6	2	2.1	P 21 21 21	A	CAMP-DEPENDENT PROTEIN KINASE CATALYTIC SUBUNIT ALPHA 2
